# Prediction and identification of synergistic compound combinations against pancreatic cancer cells

**DOI:** 10.1016/j.isci.2021.103080

**Published:** 2021-09-03

**Authors:** Yasaman KalantarMotamedi, Ran Joo Choi, Siang-Boon Koh, Jo L. Bramhall, Tai-Ping Fan, Andreas Bender

**Affiliations:** 1 Centre for Molecular Informatics, Department of Chemistry, University of Cambridge, Lensfield Road, Cambridge CB2 1EW, UK; 2 Department of Pharmacology, University of Cambridge, Tennis Court Road, Cambridge CB2 1PD, UK; 3 Cancer Research UK Cambridge Institute, University of Cambridge, Cambridge CB2 0RE, UK

**Keywords:** Molecular biology, Computational bioinformatics, Cancer systems biology

## Abstract

Resistance to current therapies is common for pancreatic cancer and hence novel treatment options are urgently needed. In this work, we developed and validated a computational method to select synergistic compound combinations based on transcriptomic profiles from both the disease and compound side, combined with a pathway scoring system, which was then validated prospectively by testing 30 compounds (and their combinations) on PANC-1 cells. Some compounds selected as single agents showed lower GI50 values than the standard of care, gemcitabine. Compounds suggested as combination agents with standard therapy gemcitabine based on the best performing scoring system showed on average 2.82–5.18 times higher synergies compared to compounds that were predicted to be active as single agents. Examples of highly synergistic in vitro validated compound pairs include gemcitabine combined with Entinostat, thioridazine, loperamide, scriptaid and Saracatinib. Hence, the computational approach presented here was able to identify synergistic compound combinations against pancreatic cancer cells.

## Highlights

• We developed a computational approach to predict synergistic compound pairs
• The approach uses transcriptional data and pathway information for scoring
• The predicted combinations with gemcitabine were validated in vitro
• Predicted combinations versus single agents had 2.82–5.18 times higher synergies

## Graphical abstract

**Figure:**
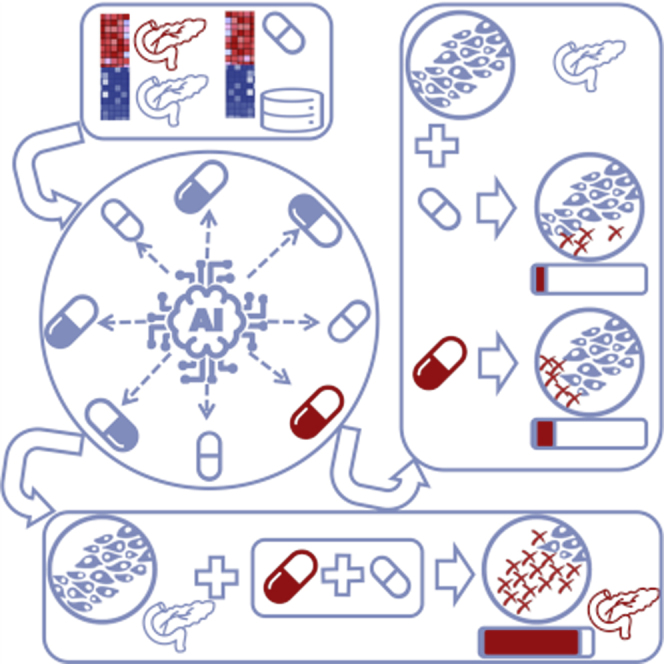

## Introduction

Pancreatic cancer is one of the most aggressive human malignancies that is commonly diagnosed only at an advanced stage (Li et al., 2004; Stathis and Moore, 2010). Gemcitabine, a nucleoside analogue of cytidine, is frequently used for treatment of pancreatic cancer, alone and in combination with nab-paclitaxel, as a first line treatment in patients with unresectable adenocarcinoma of the pancreas (Vogl et al., 2019). However, the efficacy of gemcitabine is low, with a survival rate after 12 months of only 18% (Burris et al., 1997; WasifSaif, 2006;Sultana et al., 2007). Drug combinations have been studied before for pancreatic ductal adenocarcinoma (the most common type of pancreatic cancer) to sensitize the cells to the effect of gemcitabine, increase efficacy of therapy and consequently improve survival rate (Jung et al., 2017; Moore et al., 2007; Sultana et al., 2008; Tu et al., 2017; Yachida and Iacobuzio-Donahue, 2013). Compound combinations may hence provide a treatment option with increased efficacy and lower toxicity by targeting several dysfunctional pathways at lower doses while also potentially reducing the likelihood of drug resistance (Mueller et al., 2009).

Methodologically, there are various ways of measuring synergy (Meyer et al., 2020; Vlot et al., 2019) and the choice of synergy metric directly influences the interpretation of the combinatorial screen. With respect to the data side, large combinatorial screening datasets have recently been published, such as the Merck combinatorial screen (O’Neil et al., 2016) with 22,737 experiments of 583 double combinations against 39 different cancer cell lines and the NCI ALMANAC(Holbeck et al., 2017) with 5,000 pairs of FDA-approved cancer drugs against a panel of 60 well-characterized human tumor cell lines. Various combination screenings have been integrated in resources such as DrugComb (Zagidullin et al., 2019) with 437,932 pairs. However, while cost and effort have been high for generating such data, it is clear that even those currently largest datasets cover drug chemical and cancer biological spaces only very partially. Hence, for exploring both chemical and biological spaces efficiently when exploring the potential of combination therapies, they need to be at least complemented with computational approaches. These approaches can be based on experimental screening data and features from the ligand (chemical) side (Preuer et al., 2017; Zhang et al., 2021), gene (Jeon et al., 2018; Kalantarmotamedi et al., 2018; Regan-Fendt et al., 2019), combination of gene expression and chemical features (Zhang et al., 2021), and pathways or biological networks(Gu et al., 2015; Li et al., 2018) as have been reviewed in recent articles(Bulusu et al., 2015). However, one limitation of machine learning based methods is that large scale data of compound combination screens, of preferably even the same cancer type, is required in the first place to be able to train a model on the data.

Given that available combination screening data for pancreatic cancer is limited, it would be very helpful in practice to be able to predict compound combinations based on monotherapy data alone. Large scale gene expression data of monotherapy of compounds on cancer cell lines is available in databases such as Connectivity Map (CMap) (Lamb et al., 2006) and LINCS (Subramanian et al., 2017), and this data has been successfully used in the past for transcriptional drug repositioning of single agents in several studies (Jahchan et al., 2013; Landreville et al., 2012; Wei et al., 2006). The underlying hypothesis for matching single agent drug treatments to diseases is that if the transcriptional responses of a compound is the *reverse* of a disease gene expression profile that compound has a therapeutic potential for treating that particular disease (Lamb et al., 2006). In other words, it is expected that compound treatments that restore gene expression patterns of a disease to its norm will also restore the physiological markers of the disease to the baseline levels (Wagner et al., 2015). Several methods have emerged for such a type of analysis, most of which involve finding anticorrelation of gene signatures of compounds and a disease of interest based on the above principle (Iorio et al., 2012; KalantarMotamedi et al., 2021; Lamb, 2007; Sirota et al., 2011).

Recent studies have attempted to hypothesize, based on transcriptional data of single agents, which compounds are likely to be synergistic in combination. Approaches can be categorized in methods that take into account similarity of signatures of compound treatments based on gene level (Bansal et al., 2014; Huang et al, 2014, 2019; Liu and Zhao, 2016; Stathias et al., 2018), target level (Regan-Fendt et al., 2019; Yang et al., 2019) and pathway level (Xu et al., 2018). This finding was also confirmed in DREAM challenge of synergy prediction (Hsu et al., 2016). Methods that take into account high similarity of signature of compound signatures have been successfully and more extensively validated than other approaches. This included an earlier Dream challenge winner (Bansal et al., 2014), DrugComboRanker (Huang et al., 2014; Zheng et al., 2021a) and SynergySeq (Stathias et al., 2018) approach. In some studies, apart from similarity of signatures, further considerations have been taken into account. This includes identifying dissimilarity of compound structures (Liu and Zhao, 2016) and maximal reversal of disease signature (Huang et al., 2019; Stathias et al., 2018) as further important contributing factors in finding more synergistic combinations. Many such approaches were validated either retrospectively or prospectively successfully in several cancers such as lung cancer (Huang et al., 2014) and glioblastoma (Stathias et al., 2018). Target functional similarity is also an appealing approach for synergy prediction. This can be quantified by measuring similarity of protein targets on perturbed pathways which is useful as it is independent of LINCS data. On the other hand, SynGeNet (Regan-Fendt et al., 2019) integrates gene expression data, target information, and network pharmacology of drug and disease for this purpose. It is based on scoring single agents using Connectivity Map approach and integrating a network approach to evaluate closeness of a drug’s known targets to important melanoma targets. Moreover, pathway information is an additional important resource for synergy prediction with limited studies focusing on that. It has been suggested that inhibiting multiple modules of reactivated disease signaling pathways is a promising strategy to identify drug combinations that overcome resistance (Xu et al., 2018).

**Figure 1:**
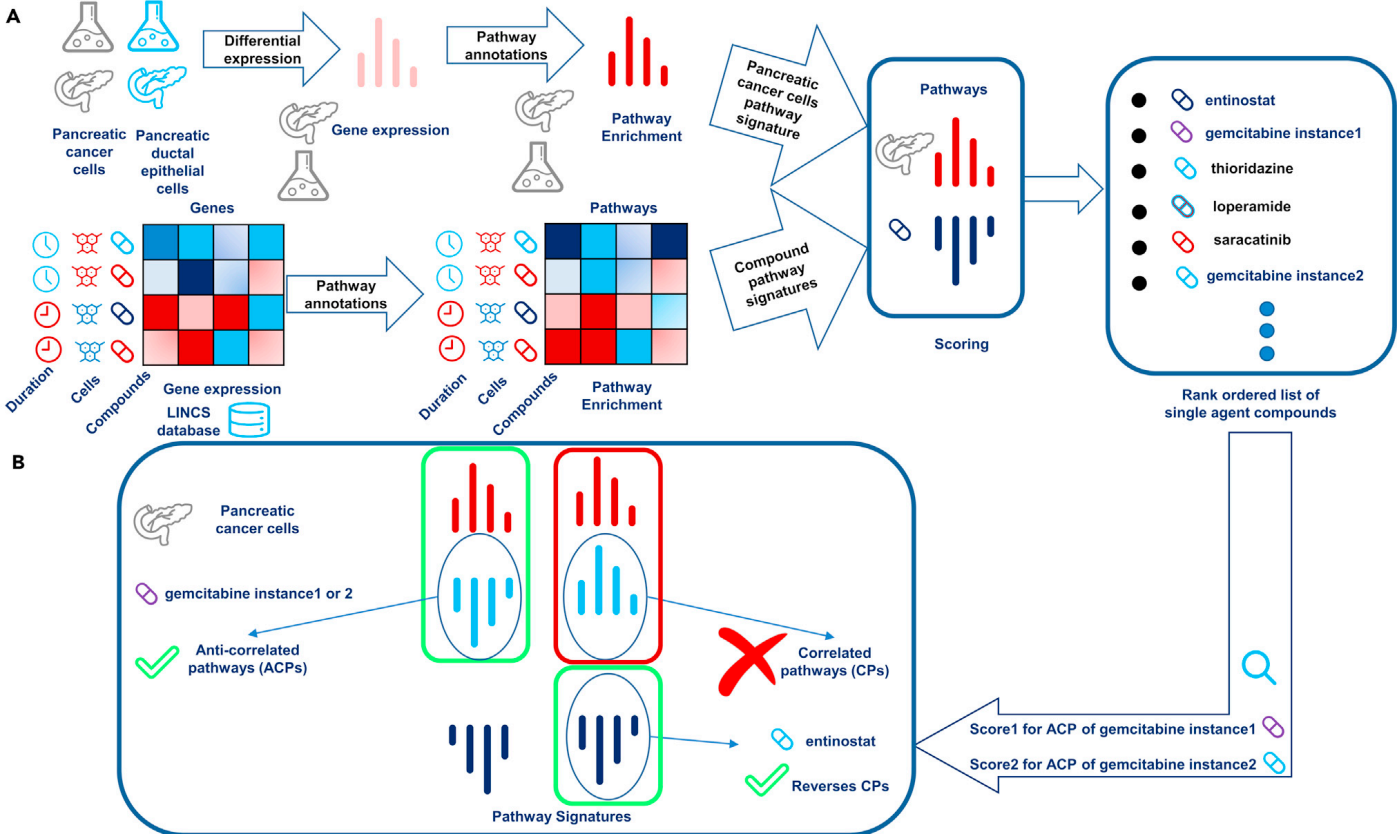
Predicting compounds active as single agents and in combination with gemcitabine against pancreatic cancer (PANC-1) cells (A) Gene expression profiles of compounds in LINCS database treated on different cell lines with different durations and pancreatic cancer cells are used as input to the method and annotated with dysregulated pathways. Next, correlation score of these pathway signatures is calculated on those pathways that are most enriched in pancreatic cancer cells. Finally, the compounds are rank ordered based on their correlation scores. (B) For predicting compound combinations, the single agent results were used and the first instance of gemcitabine in the top rank-ordered single agents was taken. Next, pathways were identified where gemcitabine instances failed to reverse the disease signature. Then, among top results, it was searched for another single agent that reversed the pathways of the first and second instances of gemcitabine. This gave rise to Score1 and Score2 and novel compounds were selected for experimental validations.

The current study now proposes, and validates for pancreatic cancer cells, a novel approach to identify potentially synergistic compound combinations from monotherapy transcriptional data. First, we have used *pathway* signatures of compounds (instead of *gene* signatures) because pathway signatures are more robust and comparable across cell lines (Wang et al., 2019). Second, we have introduced a novel hypothesis about which types of pathway dysregulation potentially leads to compound synergy. This has been achieved via a two-step process (see Figure 1). In the first step, we identified sets of pathways that are dysregulated in the PANC-1 cell line compared with pancreatic ductal epithelial cells. Then, we hypothesized that targeting the dysregulated pathways of the disease efficiently would result in identifying compounds with the desired disease-modulating effect on PANC-1 cells (Figure 1A). As gemcitabine (a current main therapy of pancreatic cancer) was identified in the first step, we then were able to elucidate the mechanistic action of gemcitabine on the transcriptional level and identify important pathways of the PANC-1 disease signature that the gemcitabine instance in LINCS database reverses (anticorrelated pathways; ACPs) and those pathways where it does not reverse and have correlation of pathway signature with disease signature (correlated pathways; CPs)*.* We next hypothesized that the CPs were the set of pathways that were contributing to gemcitabine resistance in PANC-1, and hence found a matching second drug in the database that would target preferably those pathways in the desired manner (i.e., with anticorrelation to the disease signature). This gave rise to the identification of two scores, termed Score1 and Score2, related to the first and second instances of gemcitabine in the database (Figure 1B). Moreover, pathways that were in the CP pathway set, and which were specifically important in PANC-1 compared to other pancreatic cancer cell lines were subsequently identified, giving rise to the Res-score (Resistance Score of the PANC-1 cell line). Based on those three scores–Score1, Score2, and Res-score–and some selected pathways, 30 selected candidate compounds were experimentally validated *in vitro* as single agents and in combination with gemcitabine, with methodological details and results as described in the following section.

## Results

### Prediction and experimental validation of single compounds against PANC-1 cells

Using the transcriptional drug repositioning approach described in STAR Methods we interrogated gene expression profiles of 20,413 compounds in LINCS (Subramanian et al., 2017) which were applied to 77 different cell lines using the disease gene expression profiles from the PANC-1 pancreatic cancer cell line (Gysin et al., 2012), compared with human pancreatic ductal epithelial cells. Among the highest scoring compounds, two instances of gemcitabine were ranked 11^th^ and 291^st^ among 201,776 signatures in the LINCS database, which serves as a retrospective validation of the approach (given gemcitabine is used in the clinic against pancreatic cancer). For prospective validation, candidate compounds were identified that were predicted to have growth inhibition effect in the PANC-1 pancreatic cancer cell line, which was subsequently validated experimentally in PANC-1 growth inhibition assays. Table 1 lists the criteria for the selection of each compound along with their experimentally derived GI_50_ and GI_90_ values. GI_90_ values were included as PANC-1 is a highly resistant cell line. Among the 29 compounds which have been predicted to inhibit growth of PANC-1 cells, 18 (58%) showed GI_50_ values below 10 μM (see Table 1). Among those, BMS-387032 (GI_50_ = 114nM, GI_90_ = 218nM), teniposide (GI_50_ = 546nM, GI_90_ = 4,371nM) and actinomycin D (GI_50_<1nM, GI_90_ = 4nM) were active in nanomolar concentrations and low GI_90_ values. (We do not propose all of these compounds as potential therapies, but they were found to be true positives purely in the context of the hypothesis we set out to validate.) For comparison purposes, the clinically used pancreatic cancer drug, gemcitabine, exhibited a GI_50_ of 152nM, but no GI_90_ value because it does not reach 90% inhibition even at maximal tested concentrations in PANC-1 cells. Hence, the algorithm presented here was successful in identifying active single agents in the first part of the validation performed in this study.

**Table 1:** Prediction and experimental results for selected single agents (‘S’) and compound combinations (‘C’) according to the different synergy hypotheses (Score1, Score2, Res-score and selected)

Compound	Cell line	Single/comb	Score type	Comb score	Single score	GI50 nM.	GI90 nM	Loewe	Bliss	ZIP	ZIP pValue	HSA	HAS pValue	Loewe	Loewe pValue	Bliss	Bliss pValue	Ic501 nM	Ic502 nM	Css
		Prediction score				GraphPad prism		Combenefit		SynergyFinderplus										
Semagacestat	PANC-1	C	PC		NA	>10,000	>10,000	27.40	8.90	−3.37	0.24	3.69	0.53	3.00	0.62	−3.99	0.48	44.33	9.00	32.78
Gemcitabine	PANC-1	S	PC		−0.45	152	>10,000	3.40	0.00											
Scriptaid	PANC-1	S&C	Res	−0.89	−0.33	3218	>10,000	33.30	10.40	−1.67	0.57	5.83	0.53	5.00	0.53	−2.92	0.67	10,000.00	28.47	61.35
Tacedinaline	PANC-1	S&C	Res	−0.84	−0.39	>10,000	>10,000	26.50	8.00	−1.16	0.85	3.71	0.77	2.88	0.82	−1.96	0.84	5851.86	8.60	37.61
Salmeterol	PANC-1	S&C	Res	−0.84	−0.33	4248	>10,000	14.20	2.10	−5.09	0.08	3.40	0.52	2.88	0.55	−5.49	0.28	5979.96	12.46	34.17
Triclosan	PANC-1	C	Res	−1.00	−0.28	>10,000	>10,000	13.10	5.20	1.51	0.60	2.48	0.78	2.54	0.77	1.15	0.84	0.00	14.69	20.96
Entinostat	PANC-1	S&C	Res/Score1	−0.78	−0.45	11,007	16,626	51.50	26.70	11.17	0.10	22.01	0.12	22.17	0.12	11.13	0.19	20,000.00	13.63	73.02
Entinostat	HPAF2	NA								−6.91	0.16	−1.37	0.81	−1.84	0.77	−7.83	0.16	20,000.00	3.58	66.70
Entinostat	K8484	NA								−1.28	0.76	0.08	0.99	−1.92	0.78	−3.66	0.59	5921.93	1.78	71.90
Entinostat	MIA PaCa2	NA								1.27	0.78	1.15	0.89	−1.17	0.86	−0.20	0.97	1000.00	4.13	66.08
Entinostat	TB32048	NA								−1.05	0.76	2.29	0.76	−1.09	0.81	−1.82	0.66	4734.84	3.97	77.38
Saracatinib	PANC-1	S&C	Score1	−0.66	−0.33	>10,000	>10,000	40.00	22.10	5.06	0.32	7.99	0.52	6.97	0.56	4.59	0.52	10,000.00	20.29	43.58
Thioridazine	PANC-1	C	Score1	−0.73	−0.21	9318	16,163	34.10	20.30	5.79	0.30	13.07	0.21	12.54	0.22	5.44	0.38	10,000.00	24.32	58.56
Loperamide	PANC-1	S&C	Score1	−0.71	−0.36	3200	>10,000	23.20	12.10	11.39	0.06	19.77	0.02	18.05	0.03	11.35	0.11	10,000.00	11.48	56.55
RS-17053	PANC-1	S&C	Score1	−0.83	−0.39	3154	5275	1.60	0.50	−3.10	0.54	2.76	0.77	0.51	0.96	−4.22	0.55	4307.93	11.95	60.92
TW-37	PANC-1	C	Score2	−0.81	−0.26	372	2376	15.10	1.20	−3.23	0.63	4.93	0.63	3.43	0.72	−3.77	0.65	733.39	12.03	61.11
Digoxin	PANC-1	C	Score2	−0.83	−0.21	25	66	6.70	5.30	−1.09	0.83	3.31	0.63	1.77	0.77	−1.57	0.82	30.38	18.88	49.13
Maprotiline	PANC-1	S&C	Selected CM		−0.35	>10,000	>10,000	12.10	5.30	−2.27	0.65	2.62	0.71	2.39	0.74	−3.32	0.58	10,000.00	6.26	32.32
Racecadotril	PANC-1	S&C	Selected CM&FoM		−0.36	>10,000	>10,000	31.30	24.00	5.28	0.23	6.93	0.31	6.51	0.38	5.47	0.25	202.42	2.34	29.39
Y-134	PANC-1	S&C	Selected CM&SS		−0.41	>10,000	>10,000	18.40	6.00	−1.42	0.77	5.19	0.60	3.70	0.71	−2.10	0.73	10,000.00	17.91	37.19
Dibenzazepine	PANC-1	S&C	Selected HS&FrM		−0.45	8975	13,108	20.60	7.30	1.85	0.71	8.53	0.27	7.71	0.42	2.12	0.72	884.54	5.86	58.47
Palbociclib	PANC-1	S&C	Selected MK&AB&HS		−0.45	6285	>10,000	7.00	2.90	−0.92	0.93	6.91	0.55	5.47	0.65	−1.17	0.93	10,000.00	9.87	48.63
Actinomycin D	PANC-1	S			−0.31	<1	4	18.70	1.30	−2.77	0.40	3.34	0.67	2.46	0.73	−3.84	0.56	1.26	20.55	60.58
L-168	PANC-1	S			−0.28	>10,000	>10,000	17.20	5.00	−6.55	0.22	−0.49	0.95	−1.20	0.90	−8.12	0.12	10,000.00	18.69	42.31
Clofarabine	PANC-1	S			−0.40	>10,000	>10,000	15.40	8.40	−0.30	0.97	4.70	0.46	3.83	0.63	0.40	0.97	3472.36	5.37	33.33
BX-795	PANC-1	S			−0.40	1619	9207	13.80	0.30	2.95	0.68	9.43	0.42	8.36	0.45	3.06	0.78	1258.68	24.86	48.93
Teniposide	PANC-1	S			−0.45	546	4371	11.60	1.80	−6.96	0.20	2.12	0.73	0.76	0.90	−7.10	0.28	314.06	14.53	56.14
Ciclopirox	PANC-1	S			−0.40	1002	1134	11.00	3.90	2.48	0.69	9.57	0.25	7.04	0.41	2.55	0.77	1292.55	28.12	63.24
Ursolic acid	PANC-1	S			−0.16	>10,000	>10,000	11.00	2.30	−10.80	0.01	−0.96	0.94	−1.83	0.87	−12.99	0.07	2.13	12.53	33.81
Phloretin	PANC-1	S			−0.40	>10,000	>10,000	8.10	8.10	0.75	0.81	1.16	0.83	1.09	0.82	0.75	0.88	10,000.00	6.90	24.92
BMS-387032	PANC-1	S			−0.38	114	218	7.70	1.90	−7.61	0.28	−0.83	0.90	−1.29	0.86	−9.73	0.21	155.43	7.78	53.68
Serdemetan	PANC-1	S			−0.42	3094	>10,000	7.70	3.00	−0.38	0.94	8.96	0.17	5.85	0.37	−0.31	0.96	10,000.00	10.15	49.56
Leelamine	PANC-1	S			−0.31	7567	9462	7.20	3.50	−7.80	0.42	1.94	0.87	0.98	0.95	−10.75	0.20	10,000.00	14.10	52.10
STK525924	PANC-1	S			−0.43	6684	11,519	7.20	1.40	12.81	0.06	7.57	0.42	6.71	0.45	14.45	0.09	7012.23	8.15	31.38
Medroxyprogesterone	PANC-1	S			−0.36	>10,000	>10,000	2.10	0.10	−5.39	0.25	2.56	0.67	2.84	0.68	−7.24	0.26	10,000.00	6.38	30.35

Compounds that were selected to be active as single agents and not based on any synergy scoring hypothesis were marked as ‘S’ and hence did not have any synergy prediction score (Score Comb). However, these compounds were tested in combination with gemcitabine experimentally for comparison reason and synergy scores were calculated for them. Compounds that were predicted to be active in combination were marked as ‘C’. Single score is predicted score for the compound to be active as single agent on PANC-1 cells and Comb Score is predicted score for the compound to be active in combination with gemcitabine based on any of the synergy scoring hypothesis identified in Score Type. GI_50_ and GI_90_ values for each compound on PANC-1 cells were identified using growth inhibition assays *in vitro*. Leowe and Bliss synergy scores were calculated from experimental data of combination of the compound with gemcitabine using Combenefit software. ZIP, HAS, Loewe and Bliss synergy scores were calculated also using SynergyFinder Plus web tool. pvalues for each synergy metric, IC50 and cell sensitivity score for combination (CSS) is also provided based on SynergyFinder Plus tool. Compound selection criteria is discussed in the Prospective experimental validation of predicted synergistic compound combinations section in the Results. Entinostat-gemcitabine combination which was the highest synergistic pair was also tested on four other pancreatic cancer cells (HPAF2, K8484, MIA PaCa2, TB32048).

### Evaluation of synergy hypothesis on a pathway mechanistic level

We next selected compounds to be combined with gemcitabine which we predicted to be synergistic against the PANC-1 cell line. Given different instances of gemcitabine gave rise to somewhat different gene expression profiles, the synergy scores were calculated separately for the first instance (Score1), the second instance (Score2), and for the resistance profile of the PANC-1 over other cell lines (Res-score).

**Figure 2:**
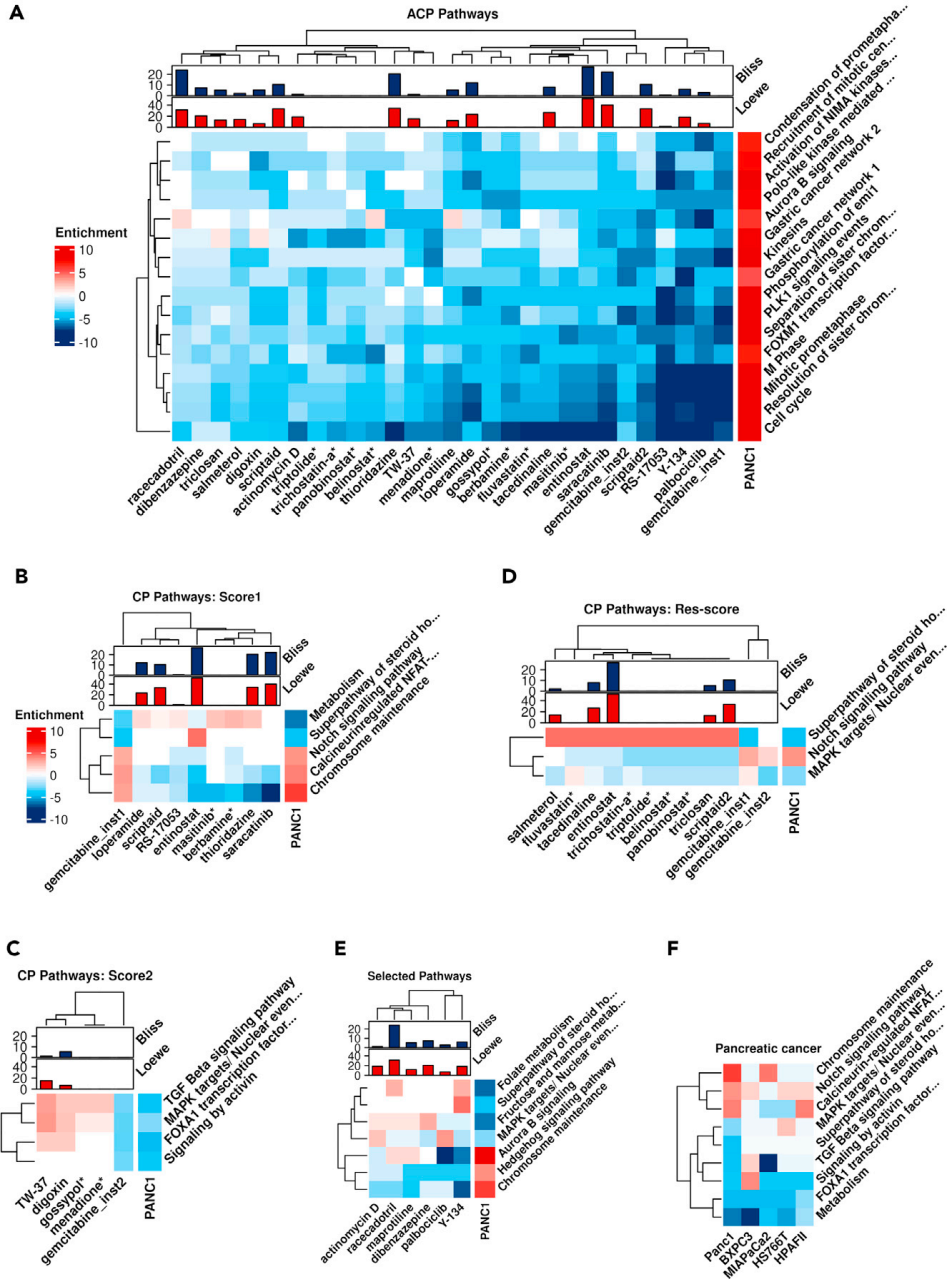
Pathway signatures of compound combinations predicted to be synergistic, compared to those of PANC-1 cells (A) Pathways where the gemcitabine signature is anticorrelated with the PANC-1 transcriptomic signature (Anticorrelated Pathways; ACPs). The second compound should also anticorrelate with these pathways from the disease signature side for having significant synergy score according to the synergy hypothesis employed in this work. Compounds marked with ∗ are shown as they had retrospective validation and the rest of the compounds were tested experimentally. Bliss and Loewe synergy scores are shown where experimental data was generated in this work for combination of the compound with gemcitabine in PANC-1 cells. (B) Pathways where the gemcitabine signature for instance1 is correlated with the PANC-1 cells signature (Correlated Pathways; CPs). The second compound should anticorrelate with these pathways (and, hence, counteract the undesired part of transcriptional dysregulation introduced by the compound) for synergy to be observed. Numbers are z-scores calculated after enrichment analysis. (C) CPs of second instance of gemcitabine and how these are reversed by other selected compounds. (D) Gene expression of pancreatic cancer cells on set of CP pathways for both instance 1 and 2 of gemcitabine are compared. (E) A few pathways were selected, and compounds based on reversal of each of these selected pathways were selected. (Color coding of enrichment scores are consistent in all heatmap plots. Pathway signatures are based on pathway enrichment scores calculated for compounds in LINCS.) (F) Pathways used for calculating Score1, Score2 and Res-score are presented for five pancreatic cancer cell lines.

The first instance of gemcitabine was ranked 11^th^ against the disease query, which was derived using a concentration of 80nM applied on A375 cells for 6 h. The second instance of gemcitabine was ranked 291^st^, for a concentration of 37nM, which was applied on MCF7 cells for 24 h. Given the large number of 201,776 profiles available, this represents the current therapy being ranked in the top 0.15%. For the first instance of gemcitabine and using *Score1*, we observed (Figure 1B) that it, as intended, reversed many enriched pathways (ACPs) in the PANC-1 pancreatic cancer cell line, including (using NCBI BioSystems(Geer et al., 2010) annotations) PLK1 signaling events, Resolution of Sister Chromatid Cohesion, Kinesins, Cell Cycle, Phosphorylation of Emi 1, and the Hedgehog Signaling Pathway (Figure 2A; Table S1). However, the transcriptomics signature of the compound was showing an (undesired) *correlation* with the disease in five other pathways (Correlated Pathways; CPs), namely Notch signaling, Superpathway of steroid hormone biosynthesis, Calcineurin-regulated NFAT-dependent transcription in lymphocytes, Chromosome Maintenance, and Metabolism pathways (Figure 2B). Among these pathways, Notch signaling has been previously identified by literature for its importance in gemcitabine resistance mechanisms, consistent with our analysis (Wang et al., 2009). Based on the synergy hypothesis formulated above, these pathways were hypothesized to be the CPs of the first instance of gemcitabine, *which hence needed to be reverted by a second compound* to achieve synergy, and to desensitize PANC-1 cells to gemcitabine treatment.

For the second instance of gemcitabine used for the calculation of *Score 2*, we observed an undesired correlation of the following pathways with the PANC-1 signature (CPs): FOXA1 transcription factor network, MAPK targets/Nuclear events mediated by MAP kinases, TGF Beta Signaling Pathway, and Signaling by Activin. Among these selected pathways, MAPK has previously been identified to be related to gemcitabine resistance mechanisms (Figure 2C)(Fryer et al., 2011). Hence *Score2* rank orders compounds based on anticorrelation to the above subset of the transcriptomic signature of disease, which was not yet sufficiently attenuated by gemcitabine applied in isolation.

As for the calculation of the *PANC-1 specific Res-score*, we aimed to identify compounds to be paired with gemcitabine that show synergy specifically in the PANC-1 cell line, as it is known to be more resistant to gemcitabine therapy than the BXPC3, Mia Paca-2, HPAFII and HS766T cell lines (Espey et al., 2011; Fryer et al., 2011). For this purpose, first, CPs of gemcitabine instances 1 and 2 were identified, which represent part of the transcriptomic signature that we deemed to be related to resistance (as above for the *Score1* and *Score2* scores). Second, pathways that were specifically dysregulated in the PANC-1 signature, compared to the other pancreatic cancer cell lines BXPC3, Mia Paca-2, HPAFII, and HS766T, were selected (Figure 2F), which were hence hypothesized to be of more relevance for resistance of PANC-1 to treatment, compared with the other cell lines (‘resistance pathway signature’). This pathway set included (according to NCBI BioSystems) the Notch signaling pathway, the Superpathway of steroid hormone biosynthesis, and MAPK targets/Nuclear events mediated by MAP kinases. Five compounds were selected based on reversal of these three pathways (Figure 2D). Moreover, five compounds were selected based on only targeting a few of pathways in the pathway sets above (Figure 2E) termed as selected score. All shortlisted compounds based on each scoring system are listed in Table1. All pathways that contributed in each scoring system are listed in Table S2.

We can see that all three of our synergy hypotheses, according to Score1, Score 2, and Res-score, give plausible mechanistic hypotheses for the selection of compounds for pairing with gemcitabine in order to achieve synergy, namely, by targeting pathways known to be involved in resistance in this cell line.

### Compound combination selection and retrospective validation

The highest scoring compounds to show synergy with gemcitabine, according to the Score1, Score2, and Res-score as outlined above (and in more detail in STAR Methods) are listed in Table S3, with scores closest to −1 indicating highest predicted synergy. Compounds that have a high negative Score1 with literature support for efficacy in combination with gemcitabine (although this does not necessarily represent *synergy*) include berbamine (Score1 = −0.88) and masitinib (Score1 = −0.76). Berbamine is known to improve cytotoxicity of gemcitabine in pancreatic cancer cell lines (Jin and Wu, 2014), while the tyrosine kinase inhibitor masitinib sensitizes gemcitabine-refractory pancreatic cancer cell lines *in vitro* as well as in phase2 clinical trials (Humbert et al., 2010). On the other hand, for Score2, gossypol (Score2 = −0.66) and menadione (Score2 = −0.65) were exhibiting highly negative values and were also supported by literature, since administration of gossypol combined with gemcitabine has been shown previously to synergistically inhibit growth of gemcitabine-resistant pancreatic cancer cells with high BCL-2 expression (Wong et al., 2012).

Compounds with highly negative Res-score include triptolide (Res-score = −0.98 and −0.96 for two distinct instances), panobinostat (Res-score = −0.98), belinostat (Res-score = −0.98), fluvastatin (Res-score = −0.95) and trichostatin-a (−0.96). Out of those compounds, triptolide (Wang and Lin, 2013) (*in vitro*) as well as belinostat(Chien et al., 2014), fluvastatin(Bocci et al., 2005) and trichostatin-a(Donadelli et al., 2007) (both *in vitro* and *in vivo*) have previously exhibited a synergistic effect with gemcitabine in pancreatic cancer cells. Belinostat and panobinostat individually inhibited growth of 6 out of 14 pancreatic cancer cell lines, including PANC-1, in previous work (Chien et al., 2014). Trichostatin-A and gemcitabine, on the other hand, synergistically inhibited growth and induced apoptosis in four pancreatic cancer cell lines and also reduced the tumor mass to 50% of its size in nude mice xenografts (Donadelli et al., 2007). Triptolide was found to enhance apoptosis of gemcitabine on the PANC-1 and AsPC-1 pancreatic cancer cell lines *in vitro* (Wang and Lin, 2013). On the mechanistic level, Belinostat, alone and in combination with gemcitabine, also significantly decreased growth and increased apoptosis of human pancreatic cancer tumors grown in immune deficient mice (Chien et al., 2014). Additionally, fluvastatin has been shown to induce apoptosis in the MIAPaCa-2 pancreatic cancer cell line, and to enhance the effect of gemcitabine synergistically (Bocci et al., 2005). Combined administration of fluvastatin with gemcitabine on MIAPaCa-2 mouse xenografts has in a previous study almost completely suppressed and significantly delayed relapse of tumor growth (Bocci et al., 2005). Hence, we can see that there is significant literature support for the different synergy scores evaluated here, in particular for the Res-score, both on an empirical and a mechanistic level.

### Prospective experimental validation of predicted synergistic compound combinations

16 compounds predicted to show synergy with gemcitabine (according to Score1, Score2, and/or Res-score) were next selected for prospective experimental validation as listed in Table 1. An additional 13 compounds had been selected for their predicted activity as single agents, but not in combination, and were screened also in combination with gemcitabine as a baseline for comparison with the above scoring system (which is a rather high baseline, since compounds were selected for individual activity in the first instance already). We also used a Gamma secretase/Notch pathway inhibitor (semagacestat) as a positive control of synergy with gemcitabine, since Gamma-secretase inhibitors have been shown previously to be synergistic with gemcitabine in pancreatic cancer mouse models (Cook et al., 2012).

Compounds that were selected using Res-score included salmeterol, scriptaid, tacedinaline, triclosan, entinostat; Score1 led to selecting entinostat, loperamide, RS-17053, saracatinib and thioridazine; and Score2 shortlisted digoxin and TW-37. Five compounds were selected based on a relatively small number of important pathways. These included racecadotril, maprotiline, dibenzazepine, Y-134, and palbociclib. Racecadotril was selected based on its effect on the Chromosome Maintenance pathway (CM) and Folate Metabolism (FoM), while maprotiline was selected based on reversal of CM. Dibenzazepine was selected based on Hedgehog Signaling pathway (HS) and Fructose and Mannose metabolism (FrM). Y-134 was selected based on reversal of CM and Superpathway of steroid hormone biosynthesis (SS), while palbociclib was selected due to reversal of MAPK targets/Nuclear events mediated by MAP kinases (MK) pathway, its effect on the HS pathway, and strengthening the effect of gemcitabine on the Aurora B signaling (AB) pathway.

Hence, in total, 30 compounds (16 predicted to by synergistic with gemcitabine, 13 predicted to be active as single agents, and a positive control) were experimentally tested in combination with gemcitabine in pancreatic cancer cells *in vitro* to evaluate our synergy hypothesis.

### Drug combination screening

Among the 16 compounds predicted to have synergy with gemcitabine the following showed higher synergy score using the Loewe model (Vlot et al., 2019) (Table 1) with experimental data: entinostat (SUM_SYN_WEIGHTED output from Combenefit of 51.5), saracatinib (40), thioridazine (34.1), scriptaid (33.3), racecadotril (31.3), tacedinaline (26.5), loperamide (23.2), and dibenzazepine (20.6). For comparison, compounds that were predicted to be active as single agents but not show synergy with gemcitabine, when tested in combination with gemcitabine had an average synergy score of 10.15 (standard deviation of ± 4.7), and the positive control (the Gamma secretase/Notch pathway inhibitor, semagacestat, which is not active as a single agent itself) obtained a synergy score of 27.4 in the Loewe model.

We next compared which of our synergy hypotheses has the highest overall synergy to understand how gene expression data could be used best to this end, the results of which are shown in Table 2. To evaluate our method to a background distribution, we have also experimentally tested all compounds that were predicted to not be synergistic (and active only as single agents) as a control. In this relative comparison we found that compounds that scored highest with Res-score were having on average 2.60 times higher synergy using the Loewe synergy metric (pvalue = 0.04) and 3.32 times higher synergy using the Bliss synergy metric (pvalue = 0.08) compared to compounds that were predicted to be active only as single agents. Score1 was leading to 2.82 and 5.18 times higher synergies in the Loewe and Bliss synergy metrics on average, respectively (pvalues= 0.04 and 0.02), compared to predicted single agent compounds. Compounds selected using Score2 could not be evaluated using this method because the number of selected compound combinations were limited and the resulting p values were not significant. Loewe, Bliss, ZIP, and HAS synergy metrics calculated using SynergyFinder Plus(Zheng et al., 2021b) tool were also compared for combinations and single agents. It shows 4.4 times higher synergy for Score1 and 2.59 times higher synergy for Res-score using Loewe Synergy metric. 3.43 and 1.98 times higher synergy is observed using HAS for Score1 and Res-Score respectively. Score-1 and Res-score have hence both been validated with respect to their ability to select synergistic compound combinations based on the data used in this study.

**Table 2:** Comparison of the different scoring systems used for selecting compound combinations and their ability to identify synergistic compound pairs

Method	Lowe			Bliss			Loewe			Bliss		ZIP		HAS		
Software	Combenefit						SynergyFinderplus									
	AVG	TTEST	DIV	AVG	TTEST	DIV	AVG	TTEST	DIV	AVG	TTEST	AVG	TTEST	AVG	TTEST	DIV
Res-score	27.72	0.04∗	2.6	10.48	0.08	3.32	7.10	0.32	2.59	0.38	0.37	0.95	0.35	7.49	0.38	1.98
Score1	30.08	0.04∗	2.82	16.34	0.02∗	5.18	12.05	0.17	4.40	5.66	0.11	6.06	0.09	13.12	0.14	3.48
Score2	10.9	0.48	1.02	3.25	0.49	1.03	2.60	0.92	0.95	−2.67	0.90	−2.16	0.96	4.12	0.81	1.09
Selected	17.88	0.08	1.68	9.1	0.1	2.89	5.15	0.10	1.88	0.20	0.24	0.50	0.23	6.04	0.15	1.60
Single agents	10.67			3.15			2.74			−2.99		−2.27			3.77	

Synergy scores of the compound combinations to be synergistic, compared with the compounds predicted to be active as single agents are provided. AVG shows average synergy scores of all compounds selected in each scoring system category. TTEST compares significance of scores of predicted compounds in each scoring category versus predicted single agents. DIV provides ratio of synergy scores of predicted compound combinations versus single agents. Compounds that were predicted to be synergistic using the Res-score were on average 2.60 times more synergistic using the Lowe synergy metric (pvalue = 0.04), and 3.32 times more synergistic using the Bliss synergy metric (pvalue = 0.08). Score1 was also leading to 2.82 and 5.18 times higher synergies in the Lowe and Bliss synergy metrics using Combenefit software, respectively (pvalues= 0.04 and 0.02). The evaluation of the Score2 selection was non-conclusive, as only two combinations were selected, and the resulting pvalue is not significant. Hence, Score1 and Res-score are reliable scoring system for synergy prediction. Loewe, Bliss, ZIP, and HAS synergy metrics calculated using SynergyFinder Plus tool were also compared for combinations and single agents. It shows 4.4 times higher synergy for Score1 and 2.59 times higher synergy for Res-score using Loewe Synergy metric. 3.43 and1.98 times higher synergy is observed using HAS for Score1 and Res-Score. The score ratios (DIV) is not provided for Bliss and ZIP as they have negative values for single agents which represents antagonism for single agents. Because the TTEST for SynergyFinder Plus metrics does not show significant values, it is better to rely on Combenefit scores in this case.

**Figure 3:**
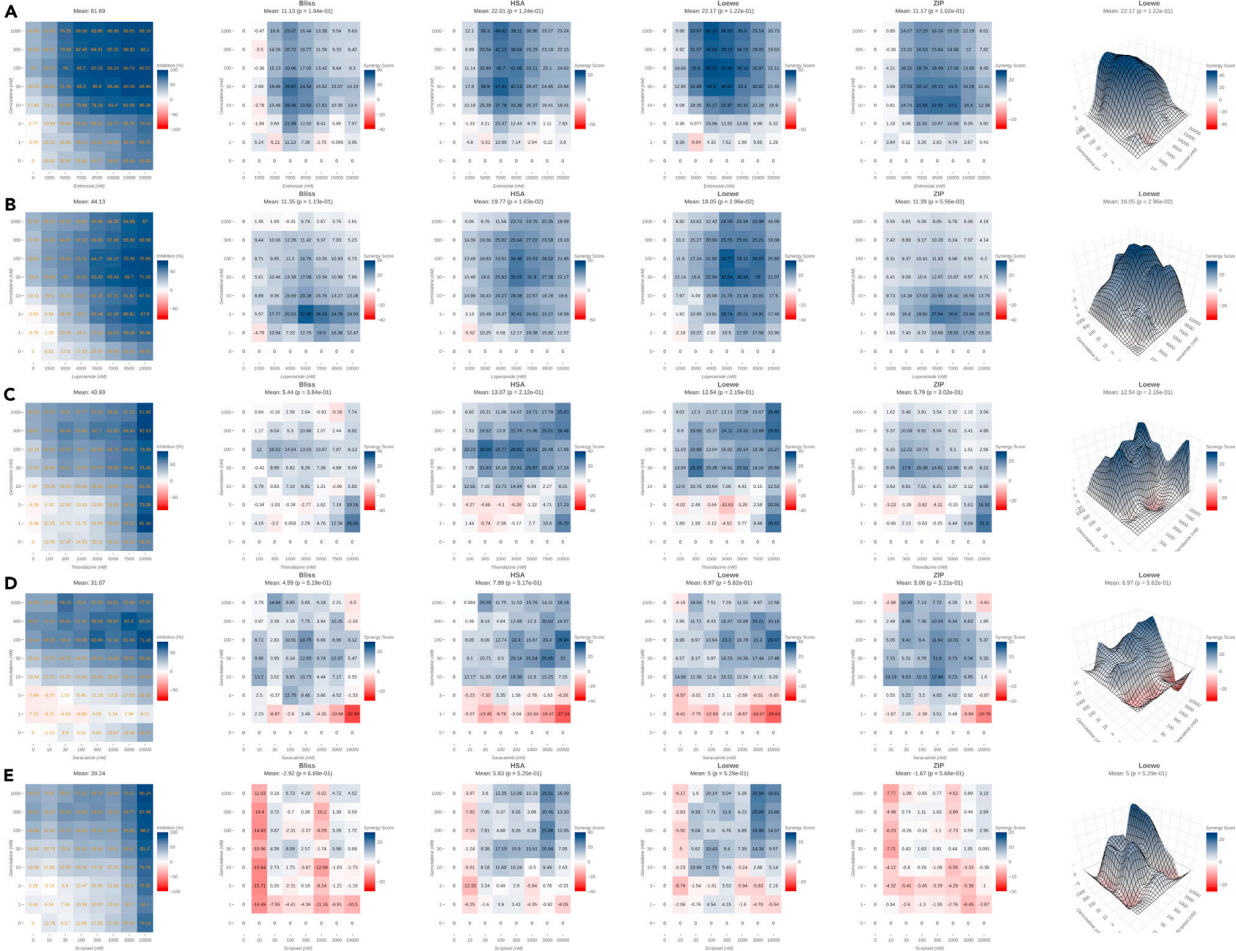
Cytotoxicity assay of the most synergistic combinations (A–E) PANC-1 cells were treated with increasing doses of gemcitabine (x-axis) versus predicted synergistic compounds (y-axis) in an 8×8 concentration checkerboard format for 24 h. Cell viability was determined by measuring the total protein content using the sulforhodamine B assays and percentage growth inhibition compared with control was provided in the matrix in the right column. From left to right dose-response, Bliss, HAS, Lowe, ZIP synergy metrics in 2D heatmap format and Loewe synergy metric in 3D format were generated using SynergyFinder web tool. Darker blue color represents high synergy for each concentration of each compound in the combination. Synergy and toxicity are presented for combination of gemcitabine with (A) Entinostat, (B) Loperamide, (C) Thioridazine, (D)Saracatinib and (E)Scriptaid. Data are represented as mean of three samples.

**Figure 4:**
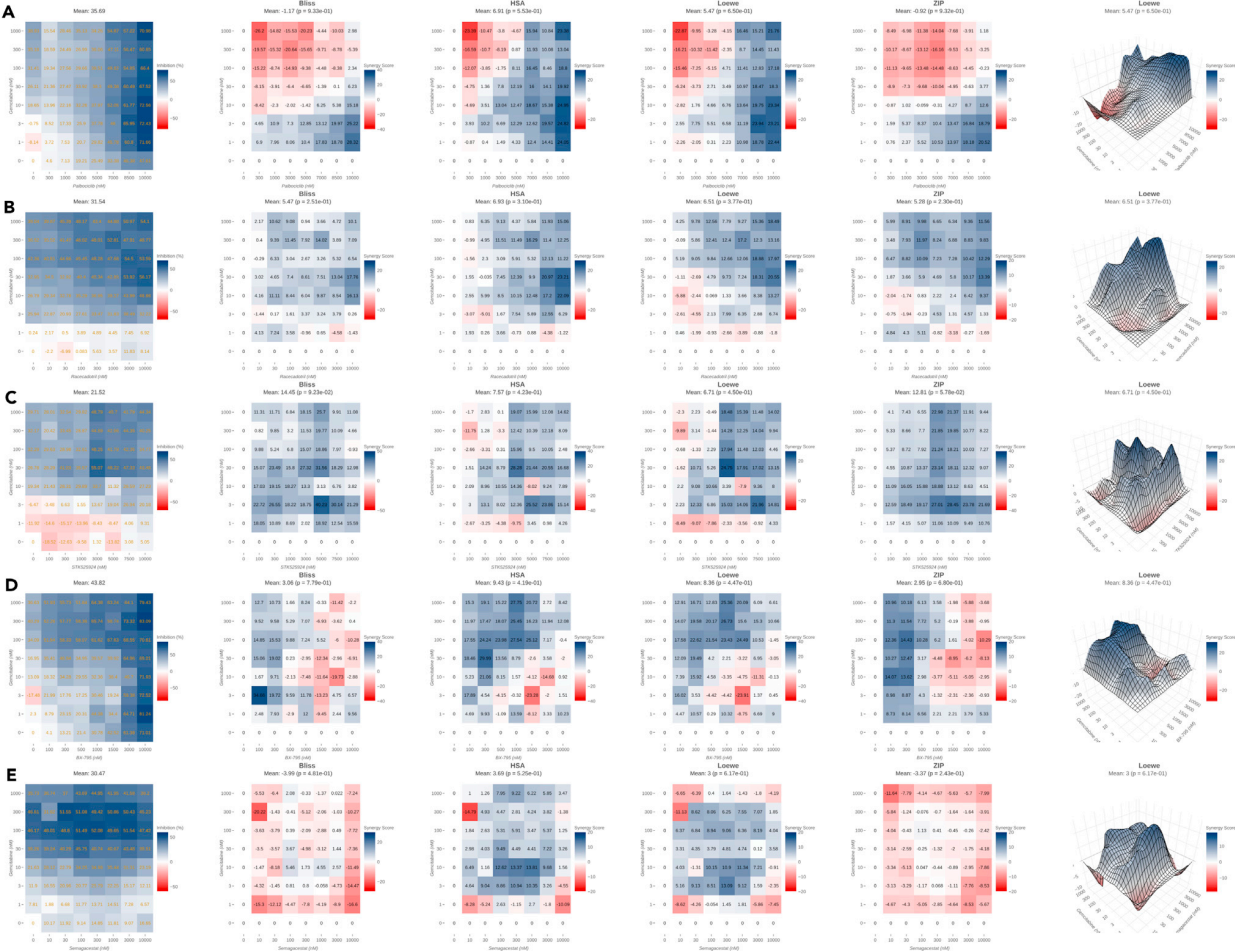
Cytotoxicity assay of the most synergistic combinations (A–E) From left to right dose-response, Bliss, HAS, Lowe, ZIP synergy metrics in 2D heatmat format and Loewe synergy metric in 3D format for gemcitabine combination with (A) Palbociclib, (B)Racecadotril, (C)STK525924, (D) BX795 and (E)Semagacestat on PANC-1 cells are visualised. Output is generated using SynergyFinder Plus webTool. Data are represented as mean of three samples.

To evaluate synergy of experimentally tested compound pairs on PANC-1 cell line, dose-response matrices, and synergy metrics for top five most synergistic compounds with gemcitabine, namely entinostat, loperamide, thioridazine, saracatinib and scriptaid are provided in Figure 3 and next five most synergistic combinations, namely palbociclib, racecadotril, STK525924, BX795, and semagacestat, are provided in Figure 4. Figures 3 and 4 compares dose-response, Bliss, HAS, Loewe, ZIP synergy metrics in 2D and Loewe in 3D for top ten most synergistic compound combinations generated using SynergyFinder Plus (Zheng et al., 2021b) web tool.

In all of these experiments, gemcitabine alone on its maximal doses reached cytotoxicity of only 46%. As mentioned before semagacestat is a gamma-secretase inhibitor and gamma-secretase inhibitors are known to be synergistic with gemcitabine in pancreatic cancer cells (Cook et al., 2012) and hence this compound was chosen as a positive control. This compound does not show any synergy with gemcitabine using Bliss and ZIP models on PANC-1 cells (Figure 4E) and just a moderate synergy in Loewe and HAS metrics. Its maximal doses increases 46% cytotoxicity of gemcitabine to only 47%. All other visualized compounds show stronger synergy than semagacestat with gemcitabine in PANC-1 cells. Entinostat shows the highest synergy levels in PANC-1 cells. However, synergy in entinostat-gemcitabine pair (Figure 3A) occures at high doses of 5000–7000nM which increases cytotoxicity of gemcitabine from 45% to 78% and 86% in these doses and at a dose of 10,000nM to 89%. Loperamide (Figure 3B) shows synergy in a wide range of doses of this compound and gemcitabine with cytotoxicity increasing from 32% to 71%. Synergy of thioridazine based on HSA metric (Figure 3C) occures in doses of 100nM and 300nM and 100nM of gemcitabine and cytotoxicity in these doses increases from 22% to 42% and 52% respectively. Its maximal doses increases cytotoxicity to 81%. Saracatinib (Figure 3D) at low dose of 300nM and 100nM of gemcitabine increases cytotoxicity of PANC-1 cells from 41% to 63% and in maximal doses cytotoxicity reaches 71%. Scriptaid (Figure 3E) is mostly synergistic in high doses of 3000–10,000nM which increases cytotoxicity of gemcitabine from 44% to 70% and 90% respectively.

Palbociclib (Figure 4A) shows synergy with gemcitabine in a wide range of doses from 300nM onwards with cytotoxicity increasing from 39% (gemcitabine alone) to 70% in its maximal doses.

Racecadotril (Figure 4B) shows moderate synergy in a wide range of doses increasing cytotoxicity from 39% to 54%. Racecadotril is inactive as a single agent with cytotoxicity of only 8% at dose of 10,000nM. STK525924 (Figure 4C) is mostly synergistic at dose of 3000nM and 30nM of gemcitabine, increasing cytotoxicity from 28% to 55%. STK525924 also as a single agent is quite inactive with cytotoxicity of only 5% on its maximal dose of 10,000nM. In case of BX-795 (Figure 4D), synergy occurs mainly on its low doses of 100–300nM with cytotoxicity increasing from 38% to 57% in doses as low as 100nM with 300nM of gemcitabine. Its maximal cytotoxicity reaches 79%. 3D views of all synergy metrics for the top 10 synergistic compounds are provided in Figures S1–S14 to complement visualization in Figures 3 and 4. Figures S1–S5 particularly shows the most synergistic compound combination gemcitabine-entinostat on PANC1, HPAFII, K8484, MIA PaCa-2, and TB32048. It is shown that this combination is only synergistic on PANC-1 cells.

**Figure 5:**
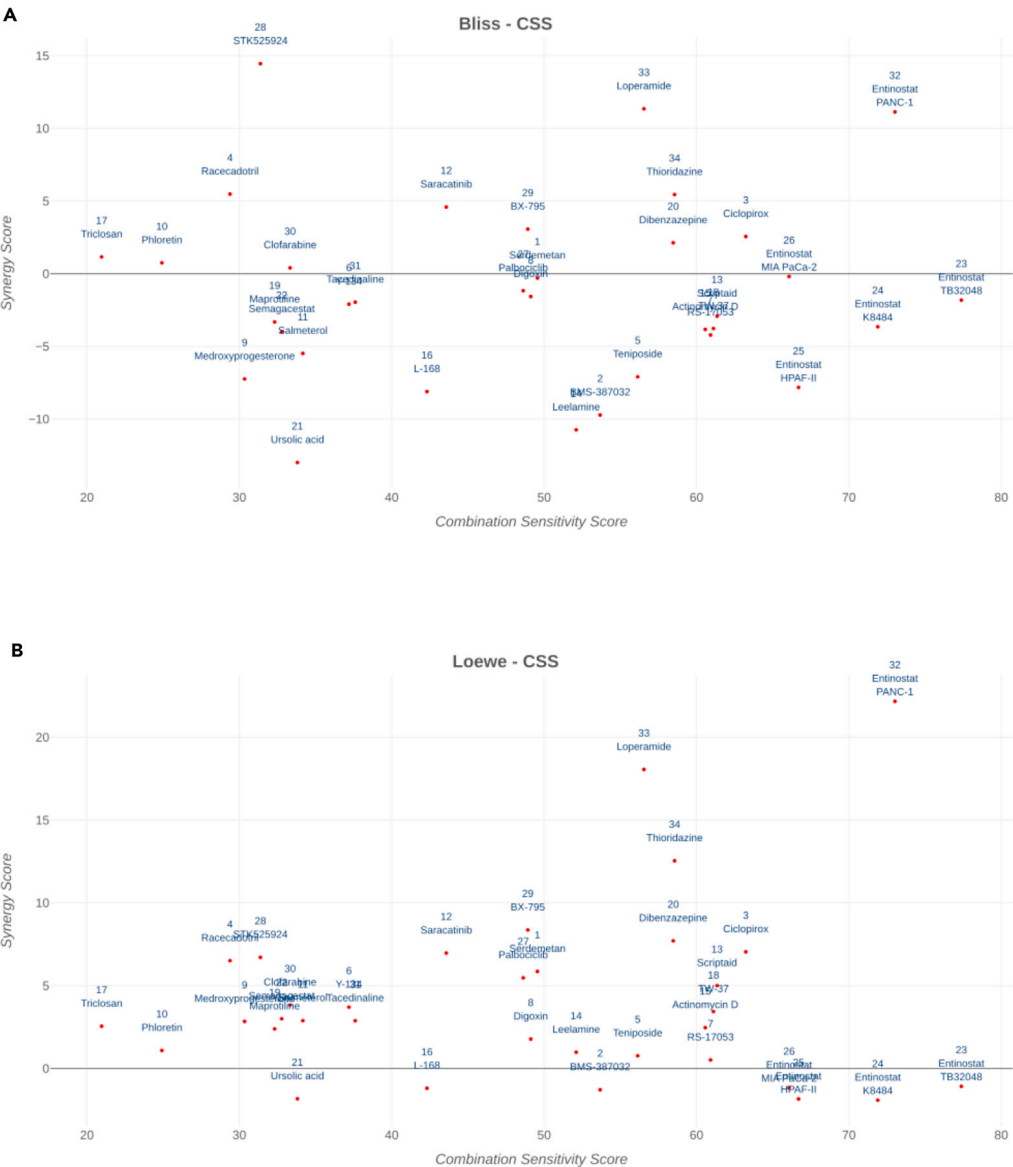
Synergy score versus sensitivity score for all experimentally validated compound combinations (A and B) (A)Bliss and (B) Loewe synergy metrics versus sensitivity score of experimentally tested compound combinations with gemcitabine was visulalised using SynergyFinder Plus web application. All pairs are tested on PANC-1 except entinostat-gemcitabine that is tested on five pancreatic cancer cell lines, namely PANC1, HPAFII, K8484, MIA PaCa-2, and TB32048, as indicated in the figure. Data are represented as mean of three samples.

To have a better overview of all tested compound combinations, synergy score (Bliss and Loewe) and cell sensitivity of all compound pairs are compared in Figure 5. HSA and ZIP metrics versus cell sensitivity are provided in Figure S15. Bliss model (Figure 5A) marks combinations of STK525924, loperamide, entinostat on PANC-1, and thioridazine as the highest synergistic compounds among which entinostat, thioridazine, and loperamide show the highest combination cell sensitivity. All entinostat-gemcitabine instances on all five mentioned cell lines show the highest sensitivity but synergy occurs only in PANC-1 cells and highest sensitivity occurs in the TB32048 cell line. Loperamide, entinostat on PANC-1, and thioridazine are marked as highest synergistic in Loewe (Figure 5B), HSA (Figure S15A), and ZIP (Figure S15B) models. Loewe (Figure 5B) and HSA (Figure S15A) models doe not mark STK525924 synergistic at all but it is highly synergistic based on Bliss (Figure 5A) and ZIP (Figure S15B) models.

The combination of entinostat and gemcitabine (Figure S16) shows the highest synergy and cell growth inhibition at sub-GI_50_ concentrations in the PANC-1 cell line (Figures 3A and S16E), but it did not show synergy in other human pancreatic cancer cell lines (MIA PaCa-2 and HPAF-II, Figures S16A and S16C) and mouse pancreatic cancer cells (K8484 and TB32048, Figures S16B and S16D). This is in agreement with the selection criterion we used for the Res-score because the aim of Res-score was to identify pathways that are specific in PANC-1 (the most resistant cell line to gemcitabine treatment) and to find synergistic combinations for this pathway set.

### Entinostat and gemcitabine act synergistically by inducing apoptosis

**Figure 6:**
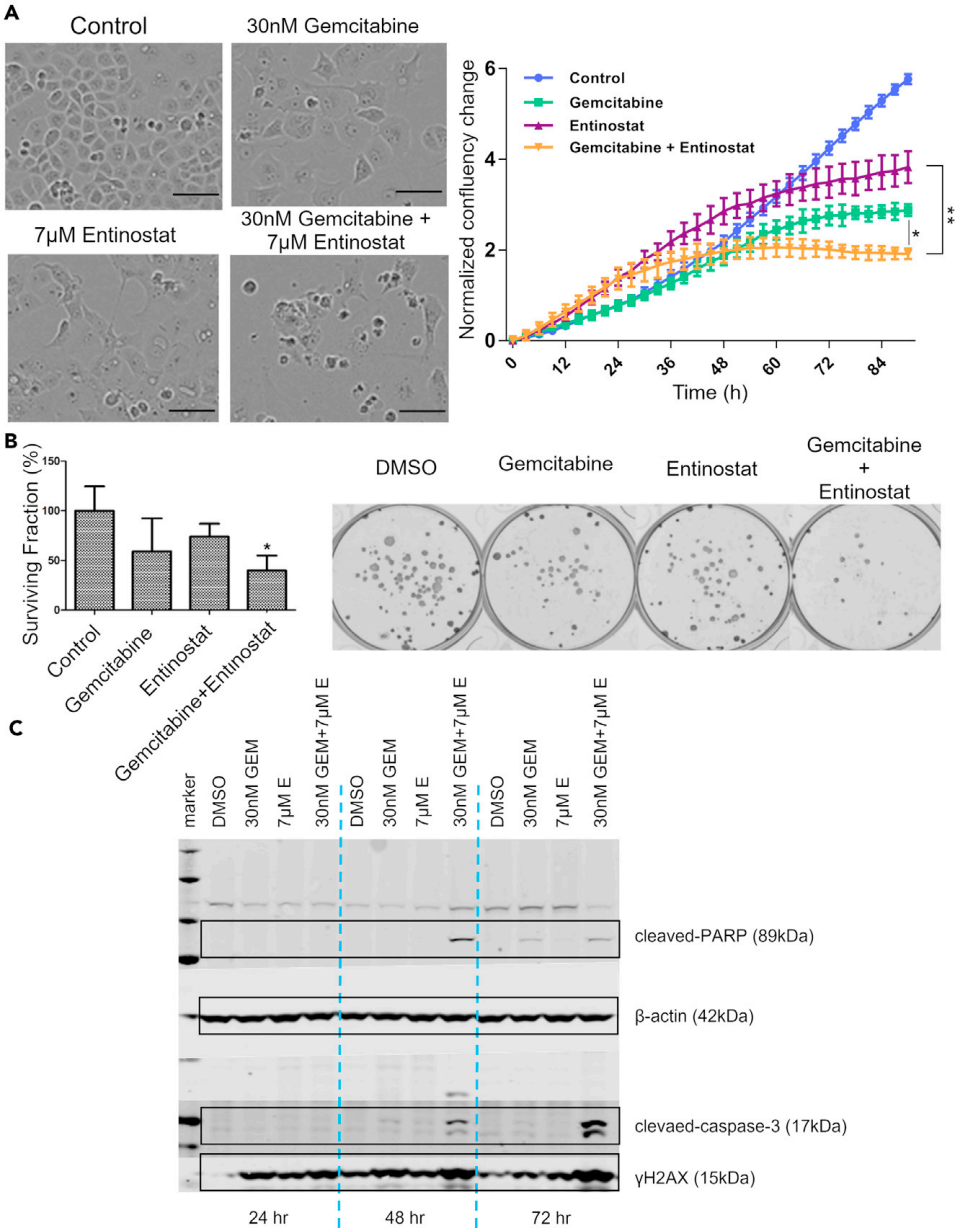
IncuCyte time-lapse imaging, clonogenic assay, and Immunoblotting for apoptosis (A) Cell proliferation in PANC-1 cells treated as indicated at synergistic concentrations (30nM Gemcitabine, 7μM Entinostat). Normalized confluency change was also measured every 3 h over a period of 84 h for each single agent and the combination. Confluency was significantly reduced in the combination group after 72 h. It was found that the growth rate was significantly reduced by the combination, compared to either single agent, with clear presence of apoptotic bodies. Data represents mean ± SD of 3 replicates, ∗ indicates p < 0.05 and ∗∗ indicates significance at p < 0.01 (based on the Kruskal–Wallis non-parametric test). Scale bar (100μm) (B) Clonogenic assays. It can be seen that the combination of gemcitabine and entinostat showed higher capacity of cells to produce progeny compared to single agent-treated groups. The number of surviving cells drops significantly in the combination compared to using each compound individually. Data are represented as mean ± SD, n = 3. ∗p ≤ 0.05. (C) PANC-1 cells were incubated with synergistic concentrations of 30nM Gemcitabine (GEM) and 7μM Entinostat (E) and total proteins were extracted after 24, 48, and 72 h for Western blotting. It can be seen that cleaved PARP and cleaved caspase 3 were elevated by the drug combination, indicating apoptosis at 48 and 72 h γH2AX, a marker of DNA damage and (later) or apoptosis was elevated by gemcitabine by 24 h but was enhanced by the combination. Protein expression of apoptotic markers, cleaved-PARP and cleaved-caspase-3 are significantly increased by the combination of gemcitabine and entinostat over time. The increase in protein expression of ɣH2AX indicates that DNA damage along with apoptosis is caused by this combination.

To understand the effects of entinostat combined with gemcitabine on the growth inhibition of PANC-1 cells, the IncuCyte system was used to obtain real time data on cell growth. It was found that the growth rate was significantly reduced by the combination, compared to either single agent (Figure 6A). Furthermore, the long-term clonogenic assays confirmed a greater inhibition in the combination than with either of the single agents (Figure 6B), and elevation of cleaved PARP, cleaved caspase 3, and γH2AX on Western blots demonstrated the induction of apoptosis by the combination (Figure 6C). Hence, we conclude that the combination of entinostat and gemcitabine acts synergistically by inducing apoptosis in a more efficient manner than either agent alone.

Entinostat in combination with gemcitabine causes increased cytotoxicity *via* complementary mechanisms, where entinostat arrests cells in the G1 phase and gemcitabine in the S phase.

**Figure 7:**
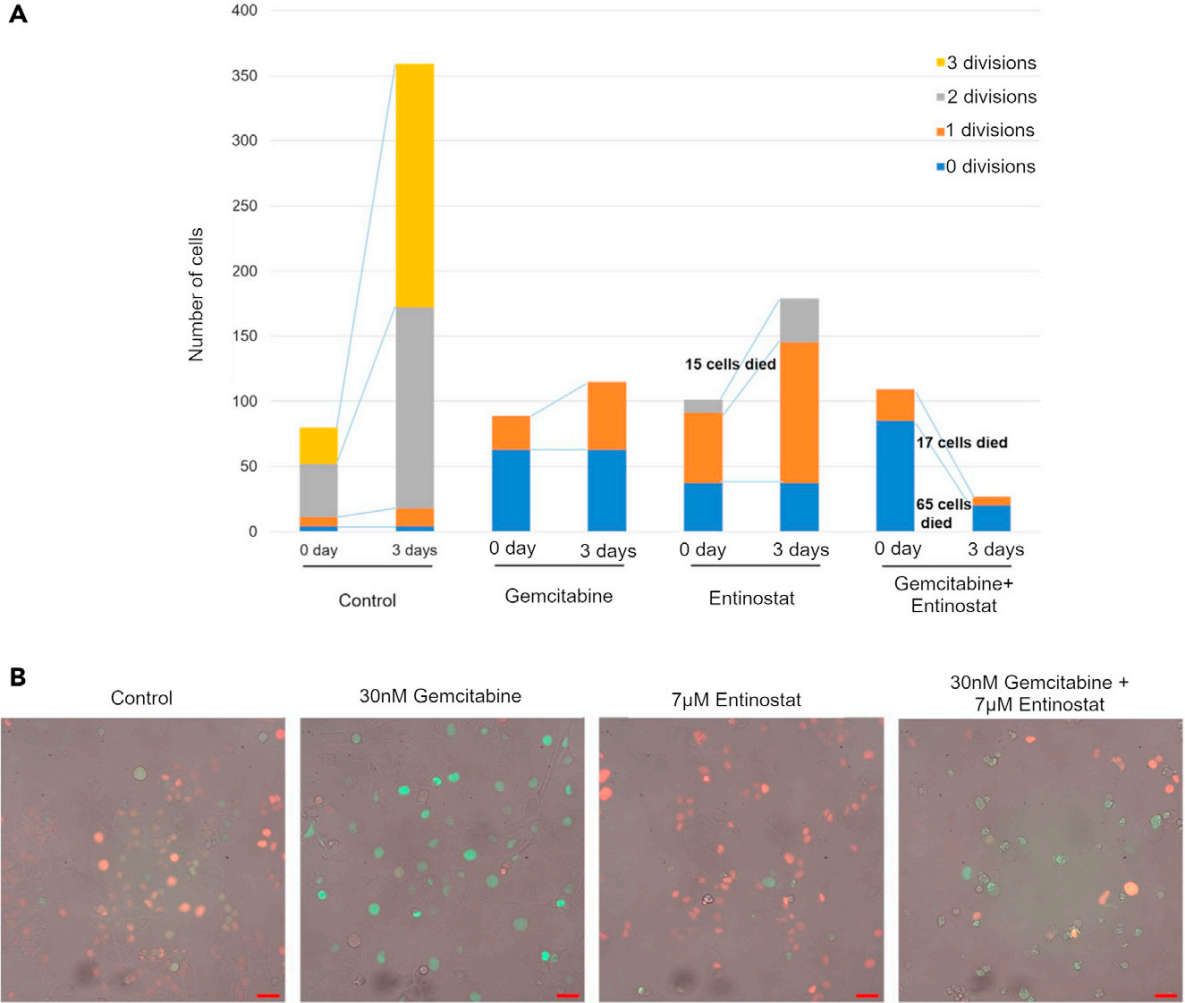
Entinostat/Gemcitabine increase cellular cytotoxicity in PANC-1 cells (A) In the control group, FastFUCCI PANC-1 cells underwent normal cell division processes. In presence of gemcitabine, S phase arrest was observed, while Entinostat blocked the G1 phase at a late time point. Fifteen cells were killed with Entinostat treatment and 64% (65 out of 102 cells) seemed to enter mitosis, but some of them failed to split at mitosis and still go back into G1. On the other hand, Gemcitabine/Entinostat dramatically increased cell death given more than 80% of cells died. Since each drug interfered at a different time of cell cycle, combination-treated cells barely survived after three days and synergy was observed. (Divisions on Day 0 was from Day 0–1, Divisions on Day 3 was from Day 0–3.) (B) Representative images of FastFUCCI PANC-1 cells treated for 72 h as indicated. S/G2-M cells (green) from G1 cells (red) based on fluorescently tagged forms of geminin and Cdt1, respectively. Scale bar, 50μm.

To study cell proliferation dynamics and effects on cell division in further detail, the FastFUCCI (Fluorescent Ubiquitination-Based Cell Cycle Indicator) system(Koh et al., 2016) and live cell imaging was used over a period of three days. In Figure 7 it can be seen that of the 80 DMSO-treated PANC-1 control cells 76 underwent one to three cell division processes, resulting in 369 cells after three days of observation. In the presence of gemcitabine, PANC-1 cells converted from the G1 phase (indicated in red, visualizing Cdt1) to the S and G2 phases (indicated in green, visualizing geminin), with the total cell number still increasing from 76 to 117, and eventually resulting in S phase arrest in agreement with earlier observations (Figure 7B; arrested green fluorescent cells) (Shi et al., 2001). On the other hand, entinostat alone was found to arrest cells in the G1 phase after at least one cell division. Only a few cells were non-viable with entinostat treatment and 64% (65 out of 102 cells) entered mitosis. Six of them failed to divide at mitosis but still proceeded into G1 phase afterward. In contrast, the combination of gemcitabine and entinostat dramatically increased cell death to over 83%. Since each drug interfered at different times of the cell cycle, combination-treated cells only in a few cases survived after a period of three days. In this section, we were able to show that different complementary mechanisms contribute to the observed compound synergy.

### Transcriptional level mechanism of action of synergistic compound pairs based on LINCS data

In order to rationalize the synergy hypotheses used for selecting compound combinations, we discuss the induced gene expression changes and pathway signatures according to LINCS data in this section in more detail for the most synergistic compounds paired with gemcitabine, namely entinostat, thioridazine, and loperamide.

Entinostat was predicted to have a highly negative Res-score and Score1, reflecting anticorrelation to the gemcitabine signature in the Res-score pathway set (the part specific to PANC-1 cells) and Score1 (the part derived from the first gemcitabine instance); see Figure 2. In the Superpathway of steroid hormone biosynthesis pathway HSD17B11 is upregulated by entinostat but downregulated by gemcitabine. When looking into the underlying data at the individual gene level, in the Chromosome maintenance pathway BRCA1, RFC5, LIG1, POLE2, PCNA are downregulated by entinostat, while RFC2, PCNA, RPA2 and LIG1 are upregulated by gemcitabine (Table S4). As opposed to the subsequent analyses, no literature evidence for those mechanistic underpinnings of synergy could be found. Mechanistically, 64% of the cells enter mitosis in the gemcitabine/entinostat combination (Figure 5), compared to 15% in the combination of gemcitabine with trichostatin-A(Gaulton et al., 2012). This is interesting to observe, in particular, given it is known that entinostat inhibits HDAC1 to a *lesser* extent (with an IC_50_ value of 510nM) than trichostatin A (IC_50_ of 20nM) (Gaulton et al., 2012), so based purely on HDAC1 inhibition the opposite order would be expected. We found that the entinostat transcriptional profile in LINCS reverses the CPs (correlated pathways, where the compound does not have the intended anticorrelation with the disease signature) of gemcitabine in the chromosome maintenance pathway by downregulating BRCA1, RFC5, LIG1, POLE2, and PCNA, while only PCNA and POLE2 are downregulated in the gene expression signature of trichostatin-A (Table S4). Hence, we hypothesize that the synergistic effect of entinostat with gemcitabine is not just due to HDAC inhibition and that taking systems data into account when trying to decipher compound action provides additional information over only looking at activity values against single targets.

For loperamide, another drug synergistic with gemcitabine in the PANC-1 experiments, PDGFA has been found to be upregulated in the gemcitabine signatures and downregulated in the loperamide signature. PDGFA is one of the drivers of tumor growth, angiogenesis, and metastasis formation in Pancreatic Ductal Adenocarcinoma (PDA) (Sahraei et al., 2012), and hence its downregulation plausibly contributes to the synergy observed. GADD45A is equally upregulated by gemcitabine but downregulated by loperamide. In this context, in p53 mutation positive pancreatic cancer patients, GADD45A was upregulated in patients with lower survival rate, also providing possible support for the observations in this work (Yamasawa et al., 2002).

Thioridazine downregulates RPA2, FOS, and INPP1, which are upregulated in the gemcitabine gene signature. High levels of RPA2 expression have been associated with adverse disease progression and it may also be a therapeutic potential target for treating colon cancer itself (Givalos et al., 2007), while FOS gene expression has been found to be associated with progression of pancreatic cancer tumors(Guo et al., 2015). INPP1 is highly expressed in aggressive human cancer cells and primary high-grade human tumors(Benjamin et al., 2014). Hence, thioridazine reverses the (undesired) CPs of gemcitabine on RPA2, FOS, and INPP1, which have been previously shown to be related to adverse patient treatment outcomes, also underpinning the rationale of synergy of compound combinations validated in this work.

We next compared similarity of gene expression patterns on the individual gene and pathway level. We found that on the gene level. Two instances of gemcitabine are provided in the Table S4. The upregulated genes of the two instances have 6.4% Tanimoto similarity (6 shared genes in 94 total unique genes) and the downregulated genes have 13.6% Tanimoto similarity (12 shared genes in total 88 unique genes). Hence, the gene signatures of both compounds are very different from each other. However, pathway signatures of gemcitabine instances 1 and 2 have 82.9% correlation together. This shows that using pathway signatures we get a more robust signal of specific compounds.

Hence, among the most synergistic compounds we have identified genes that show anticorrelation between transcriptomic changes induced by gemcitabine and the paired compound, providing a mechanistic rationale for those observations that in many cases is also supported by clinical evidence.

## Discussion

In this work, we presented and validated a novel systematic approach to predict the synergy of compound combinations based on transcriptional data and pathway annotations. The synergy hypotheses used here were based on the assumption that the transcriptional activity of a second compound paired with the main therapy should be *anticorrelated to the disease signature not yet reverted by the main treatment*. Thirty-one compounds were shortlisted in total, among which 13 were predicted to be active as single agents only. 16 compounds were predicted to show synergy with gemcitabine while 12 were predicted to be active both as single agents and in combination. For reference, we had one positive synergistic control (semagacestat) and one single agent positive control (gemcitabine).

Among the predicted combinations entinostat showed the highest synergy (Loewe synergy of 51.5, Bliss synergy of 26.7) with gemcitabine, which was higher than our positive control (Loewe synergy of 27.4, Bliss synergy of 8.9). The entinostat-gemcitabine combination was previously (but after the actual conductance of the current work) identified as a synergistic combination in pancreatic cancer cells (Ma et al., 2017). Additionally, further novel synergistic pairs including gemcitabine/thioridazine and gemcitabine/loperamide were identified.

While the combination of thioridazine with gemcitabine has been patented before for non-small-cell lung carcinoma (NSCLC) (Huang et al., 2016), it is novel in pancreatic cancer as suggested from this work. Thioridazine and its family member penfluridol has been shown to cause cell death in pancreatic cancer cells *via* activation of protein phosphatase 2 (PP2A) and to affect protein expression levels in cell cycle regulation, apoptosis, and multiple kinase activities (Chien et al., 2015). Thioridazine inhibits cancer stem cells (CSC) of various origins such as myeloid leukemia, glioblastoma, and lung, liver, ovarian and breast cancers (Chan et al., 2018). It has been effective *in vitro* by inhibiting CSC spheroid formation and inducing apoptosis and *in vivo* by reducing xenograft tumor volume in mice. The plasma peak concentration (C_Max_) of thioridazine after a single oral dose of 50 mg reaches 280 nM(Chigaev et al., 2015). We have shown in this work that synergy between thioridazine and gemcitabine occurs in a wide range of concentrations of both drugs, including at 100nM and 300nM of thioridazine. While after application of 300nM of gemcitabine on PANC-1 cells 60% of the cancer cells were still surviving, addition of thioridazine at a concentration of 300nM caused this to drop to 42%. Hence, thioridazine at its safe dose increases cell death of PANC-1 cells induced by gemcitabine by 18% in absolute terms (or nearly a third in relative terms), which given the PK considerations described here may also translate into clinical relevance.

Another combination with gemcitabine suggested from the current work is loperamide, which is an anti-diarrheal agent and targets the μ-opioid receptors. Loperamide has been shown to enhance the cytotoxicity of doxorubicin and reverse multi-drug resistance in breast cancer cells (Zhou et al., 2012). It has also reversed multi drug resistance to bortezomib in colon cancer cells (Kim et al., 2019). Here, we have shown that it increases cytotoxicity of gemcitabine to PANC-1 cells and shows high synergy in a wide range of doses.

Overall, the computational approach presented here has successfully predicted synergistic compound combinations for pancreatic cancer cells using the transcriptional response data of single agents and gene expression profiles of cancer cell lines. The method lends itself to mechanistic interpretation and it is potentially applicable in other cancer types and beyond.

### Limitations of the study

Predicted combinations were validated for PANC-1 cells and only the highest synergistic pair was validated on five pancreatic cancer cells (PANC-1, MIA PaCa-2, HPAF-II, K8484, and TB32048). It was also shown that this pair (Entinostat-gemcitabine) is selectively synergistic only on PANC-1 cells as expected due to the type of scoring system chosen (Res-score). As one limitation of the work, this selectivity on PANC-1 was experimentally proven only for the most synergistic combination and not the rest of the pairs.

The computational approach is limited to the compound database used here (LINCS) and cannot be extended to larger compound databases without having transcriptional data of single agents. LINCS is also limited to the 77 cell lines used for generating the data. The cell lines represent the biological space used here for measuring compound treatment effect which is not comprehensive.

## STAR★Methods

### Key resources table

**Table:** 

REAGENT or RESOURCE	SOURCE	IDENTIFIER
**Antibodies**		

cleaved PARP	Cell Signaling	Cell Signaling Technology Cat# 5625, RRID:AB_10699459
cleaved caspase 3	Abcam	Abcam Cat# ab13847; RRID:AB_443014
β-actin	Abcam	Abcam Cat# ab6276, RRID:AB_2223210
γH2AX	Millipore	Millipore Cat# 05-636, RRID:AB_309864
IRDye800CW- conjugated antibodies	LI-COR	http://www.licor.com/bio/products/reagents/irdye_secondary_antibodies/irdye_secondary_antibodies.jsp
IR680CW-conjugated antibodies	LI-COR	http://www.licor.com/bio/products/reagents/irdye_secondary_antibodies/irdye_secondary_antibodies.jsp

**Chemicals, peptides, and recombinant proteins**		

maprotiline	Sigma-Aldrich	M9651
palbociclib	Sigma-Aldrich	PZ0383
tacedinaline	Sigma-Aldrich	C0621
digoxin	Sigma-Aldrich	D6003
medroxyprogesterone	Sigma-Aldrich	M6013
loperamide	Sigma-Aldrich	L4762
salmeterol	Sigma-Aldrich	PHR1947
triclosan	Sigma-Aldrich	PHR1338
paclitaxel	Sigma-Aldrich	T7402
phloretin	Sigma-Aldrich	P7912
teniposide	Sigma-Aldrich	SML0609
racecadotril	Sigma-Aldrich	SML0043
Y-134	Tocris	2676/10
RS-17053	Tocris	0985/10
L-168,049	Tocris	2311/10
actinomycin D	Tocris	1229/10
BX-795	Selleckchem	S1274
clofarabine	Selleckchem	S1218
serdematan	Selleckchem	S1172
BMS-387032	Selleckchem	S1145
saracatinib	Selleckchem	S1006
TW-37	Selleckchem	S1121
ursolic acid	Selleckchem	S2370
gemcitabine	LKT	G1745
ciclopirox	LKT	C3208
scriptaid	Cayman	10572
entinostat	Cayman	13284
NVP-TAE684	Biovision	1683
semagacestat	Biovision	2430
BRD-A68061604 (STK525924)	Vitas M Laboaratory	STK525924
thioridazine	MP Biomedicals	15689101

**Experimental models: Cell lines**		

PANC-1, MIA PaCa-2	European Collection of Cell Cultures	N/A
HPAF-II	American Type Culture Collection	N/A
K8484 and TB32048	David Tuveson’s lab at Cold Spring Harbor Laboratory	N/A

**Software and algorithms**		

GraphPad Software	Prism	www.graphpad.com
SynergyFinderPlus	Zheng et al., 2021b	https://synergyfinderplus.org/
R	R-Project	https://www.r-project.org

### Resource availability

#### Lead contact

Further information and requests for resources and reagents should be directed to and will be fulfilled by the Lead Contact, Dr Yasaman KalantarMotamedi(yk313@cantab.net)

#### Materials availability

All unique/stable reagents generated in this study are available from the Lead Contact with a completed Materials Transfer Agreement.

Key resources including details of key reagents and cell lines used are available in the Key Resources Table.

### Experimental model and subject details

#### Cell culture and chemicals

Human pancreatic cancer cells (PANC-1, MIA PaCa-2 and HPAF-II) were obtained from either the European Collection of Cell Cultures (PANC-1 and MIA PaCa-2) or the American Type Culture Collection (HPAF-II). They were authenticated by the CRUK Cambridge Institute Biorepository core facility, using either the Promega GenePrint10 system or the Promega PowerPlex 16HS kit, and were grown in DMEM with 10% FBS (GIBCO, MA, USA). Murine pancreatic cancer cells K8484 and TB32048 were established from tumours in KRasG12D; p53R172H; Pdx1-Cre mice by members of David Tuveson’s lab at Cold Spring Harbor Laboratory (Hingorani et al., 2005; Olive et al., 2009) and were grown in DMEM with 5% FBS.

All cell lines were grown up to a maximum of 20 passages and for fewer than 6 months following resuscitation. They were routinely verified to be mycoplasma-free by the CRUK Cambridge Institute Biorepository core facility using the Mycoprobe Mycoplasma Detection Kit (R&D Systems, MN, USA). Maprotiline, palbociclib, tacedinaline, digoxin, medroxyprogesterone, loperamide, salmeterol, triclosan, paclitaxel, phloretin, teniposide and racecadotril were purchased from Sigma-Aldrich; Y-134, RS-17053, L-168,049 and actinomycin D were ordered from Tocris; BX-795, clofarabine, serdematan, BMS-387032, saracatinib, TW-37 and ursolic acid were supplied by Selleckchem. In addition to the above listed chemicals, gemcitabine and ciclopirox (LKT), scriptaid and entinostat (Cayman), NVP-TAE684 and semagacestat (Biovision), BRD-A68061604 (Vitas M Laboratory), thioridazine (MP Biomedicals) were obtained, dissolved in DMSO in aliquots of 10-30mM, kept at -20°C and used within 3 months. Final DMSO concentrations (≤0.2%) were kept constant in all experiments.

### Method details

#### Cytotoxicity assay and synergy calculation

Drug cytotoxicity in vitro was assessed by the means of Sulforhodamine B colorimetric (SRB) assay (Vichai and Kirtikara, 2006). Cells were plated in a 96 well plate and dosed with a range of concentrations of drugs (0.001 μM to 10 μM) and incubated for 72 h at 37°C. Cells were then fixed (3% trichloroacetic acid, 90 minutes, 4°C), washed in water and stained with a 0.057% SRB (Sigma-Aldrich, #230162-5G) solution in acetic acid (w/v) for 30 minutes. The plates were washed (1% acetic acid), and the protein-bound dye was dissolved in a 10 mM Tris base solution (pH 10.5). Fluorescence was measured using the Tecan Infinite M200 plate-reader (excitation 488 nm, emission 585 nm). 50% Growth Inhibition (GI_50_) values of each drug were calculated by comparing with solvent control.

For compound combination assays, cells were seeded for 24 hours in 96-well plates and then treated with a serial dilution of each agent and gemcitabine in an 8 X 8 concentration format.

The effect of the combination was analyzed by Combenefit (Di Veroli et al., 2016). The Combenefit software generates a set of synergy scores based on the Bliss (1939) and Loewe models (Di Veroli et al., 2016). The choice of synergy scores significantly influences interpretation of drug combination screens (Meyer et al., 2020; Vlot et al., 2019). Similar synergy calculation methodology is used in authors’ previous work (Koh et al., 2015).

#### IncuCyte time-lapse imaging

Images of cells were acquired with the IncuCyte Live Cell Imaging microscopy (Essen Bioscience, MI, USA) at every three hours under cell culture conditions with 10X objective. Averaged cell confluence was calculated from three random fields of view per well using the IncuCyte in-built algorithm. Relative confluence values were obtained by normalizing each value to the time zero value in each sample.

#### Clonogenic assay

Cells were plated 24 hours prior to treatment. After 48 hours of treatment, equal numbers of viable cells from each sample were reseeded in fresh medium and left to grow for a week or two depending on the cell density. Cells were then fixed with 70% methanol and stained with 0.2% crystal violet (Sigma-Aldrich, MO, USA). Colonies were imaged and quantified using the Gelcount (Oxford Optronix). Plating efficiency was calculated from the ratio of the number of colonies to the number of cells seeded. The number of colonies that arose after treatment was expressed as surviving fraction. This was derived from the ratio of the number of colonies formed after treatment to the number of cells seeded multiplied by plating efficiency of the control (Franken et al., 2006).

#### Immunoblotting

For immunoblotting, whole-cell extracts were obtained by lysis in RIPA buffer (50mM Tris pH8.0, 2mM EDTA, 150mM sodium chloride, 1% NP-40, 0.5% sodium deoxycholate, 0.1% SDS) and resolved using the SDS-PAGE gel system (Life Technologies, MA, USA). Blots were analyzed using the Odyssey Infrared Imaging System (LI-COR, NE, USA). Primary antibody cleaved PARP (#5625S) was obtained from Cell Signaling (MA, USA), cleaved caspase 3 (ab13847) and β-actin (ab6276) were purchased from Abcam (Cambridge, UK) and primary antibody γH2AX from Millipore (05-636). As secondary antibodies IRDye800CW- and IR680CW-conjugated antibodies from LI-COR were used in immunoblotting.

#### Acquisition, processing and analysis of live-cell time-lapse sequences

PANC-1 FastFUCCI cells (Koh et al., 2016) were kept in a humidified chamber under cell culture conditions. Images were taken on five fields of view per well, every seven minutes over 72 hours, using the Zeiss Axio Observer system with 10X objective. An equalization of intensities over time was then performed to each channel using the ZEN software (Zeiss, Oberkochen, Germany).

### Quantification and statistical analysis

#### Statistical analyses

Data from SRB assay were analyzed using the GraphPad Prism (Version 7) (GraphPad Software, 2015) built-in tests or the Combenefit (Di Veroli et al., 2016) software. An ordinary one-way ANOVA with a Tukey’s multiple comparisons test was performed using GraphPad Prism version 7 for Windows (GraphPad Software, CA, USA, www.graphpad.com). Data represents mean ± SD of 3 replicates, ∗ indicates p < 0.05 and ∗∗ indicates significance at p < 0.01 (based on the Kruskal-Wallis non-parametric test).

#### Gene expression data retrieval and pathway signature calculation for compounds

The compound dataset used in this project was retrieved from the LINCS database (Cheng and Li, 2016; Subramanian et al., 2017) (Phase I). LINCS at the time of this study contained gene expression profiles of a set of 20,413 compounds applied to 77 different cell lines including 59 cancer cell lines. In this work, the LINCS Application Processing Interface (Lincscloud.org, accessed 2015, replaced by clue.io today) was used to retrieve gene signatures of all compounds in the dataset, including the list of 50 most up- and down-regulated landmark genes among significantly differentially expressed genes in each cell line after each compound treatment (without taking into account the expression level). Landmarks genes were 978 genes profiled in L1000 platform that were sufficient to recover 82% of the information in the full transcriptome (Subramanian et al., 2017). In this work, gene expression of different instances of the same compound on different cell lines were not aggregated together and were treated separately.

As LINCS did not include any pancreatic cancer cell line, we used pathways instead of genes to define effect of compounds on the cell. From the NCBI BioSystems (Geer et al., 2010) database (accessed in 2015) all human biological pathways and the name of genes that belonged to those pathways were downloaded. This constituted 2,010 pathways with annotated gene members in each pathway. In this work, for each compound instance in LINCS, the number of genes up- and down-regulated in each pathway were counted (separately for each direction). To normalize the score for each compound, 20,000 random gene sets with the same length as the compound signatures (50 genes) were generated to constitute a background population. Next, z-scores were calculated for each pathway ‘p’ for each compound ‘c’ compared to the background population using the following formula:

$$Score_{c,p}=\frac{N_{c,p}-\mu_{p}}{\sigma_{p}}$$

where *N*_*c*,*p*_ denotes number of shared genes in the compound c and pathway ‘p’, $\mu_{p}$ and $\sigma_{p}$ denote average and standard deviation of number of shared pathways with pathway ‘p’ and the background population, respectively (random gene sets).

#### Gene expression data retrieval and pathway signature calculation for pancreatic cancer cell lines

We next needed to define the gene expression differences between healthy and disease (here pancreatic cancer) states. For this purpose, the gene expression profile of GEO dataset: GSE45765, containing the whole genome gene expression profile of normal human pancreatic ductal epithelial cells specimen and pancreatic cancer cell lines (Gysin et al., 2012) was imported using GenePattern (Reich et al., 2006) GEO Importer tool. Next, untreated cancer cell lines (PANC-1 and BXPC3) were each compared with the normal human pancreatic ductal epithelial cells specimen and the log_2_ fold change was calculated for each gene in each cell line (PANC-1 was used for synergy prediction and testing only). The genes were sorted based on their log_2_ fold changes and the 50 most over- and underexpressed genes constituted the disease signature for each pancreatic cancer cell line. Next, the number of shared genes between the pancreatic cancer disease signature and each of the pathways in Biosystems was counted and enrichment scores were calculated for each pathway to generate a pathway signature for each of the pancreatic cancer cell lines (per direction, analogous to the compound pathway enrichment calculation). The only difference was that for normalising pathway enrichment scores for the disease signature the random gene sets were selected from genes that were in the assay used for gene expression profiling of cancer cells (in this case the HG-U133A_2 Affymetrix Human Genome U133A 2.0 Array).

This pathway enrichment analysis led to a pathway signature for each compound in the LINCS database, and a pathway signature for each pancreatic cancer cell line, based on the 50 most up- and downregulated genes, per direction, which were annotated with NCBI Biosystems pathways.

#### Compound-disease matching

Similar to the original ‘Connectivity Mapping’ approach (Lamb et al., 2006) we were interested in compounds whose pathway signature was anticorrelated with the disease signature. To this end, the pathways with highest normalised score in the disease were identified for targeting by the compounds. Significantly up- or downregulated pathways (with a p-value<0.01, equivalent to a Z-score cut-offs of above 2.58 or under -2.58) were identified to this end. Next, the Pearson correlation of the pathway signature of the compounds in LINCS with the disease pathway signature was calculated, but only on the subset of pathways that were found to be significantly dysregulated in the diseases signature. Then, the compounds were rank ordered based on their anticorrelation scores. This rank ordered list of compounds was annotated with predicted protein targets and pathways to facilitate selection of potentially active compounds in a more informed manner. In this regard, a Naïve Bayes target prediction algorithm (Koutsoukas et al., 2013) was utilised to annotate ranked compounds with their targets, based on bioactivity data from CHEMBL v.17 comprising 385,126 compound-protein pairs, 1,643 distinct proteins and 226,791 unique compounds.

## Data Availability

• Source data and code for generating Figures 2, 3, 4, 5, and S1–S16 is available in the following GitHub repository: https://github.com/pathwayBasedDrugRepositioning/PancreaticCancer. • SynergyFinderPlus webtool was used for measuring synergy: https://synergyfinderplus.org/. • R software was used for figure generation: https://www.r-project.org/.
