# Supplementary material for: Prediction and identification of synergistic compound combinations against pancreatic cancer cells
Source: iScience. 2021 Sep 3;24(9):103080. doi: 10.1016/j.isci.2021.103080 (PMC8456050; doi:10.1016/j.isci.2021.103080)
Supplement: Document S1. Figures S1–S16 and Tables S1–S4 [file mmc1.pdf]

iScience, Volume *24*

Supplemental information

Prediction and identification of synergistic compound combinations against pancreatic cancer cells

Yasaman KalantarMotamedi, Ran Joo Choi, Siang-Boon Koh, Jo L. Bramhall, Tai-Ping Fan, and Andreas Bender

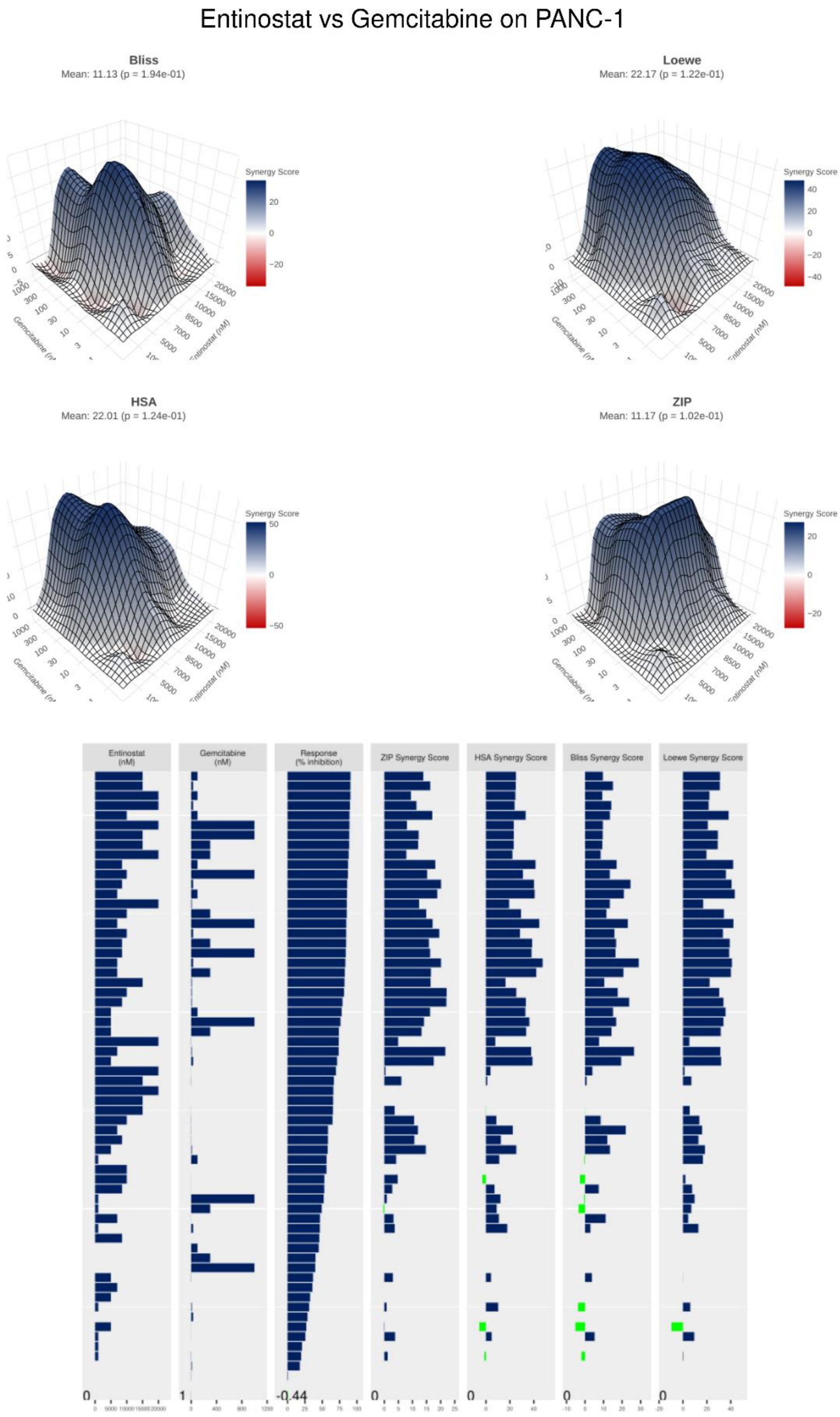

Figure S1: Entinostat vs Gemcitabine synergy on PANC-1 cells, Related to Fig. 3A. Bliss, Loewe, HAS and ZIP synergy metrics in 3D as well as bar plot are visualised. Synergy was calculated for three experimental samples.

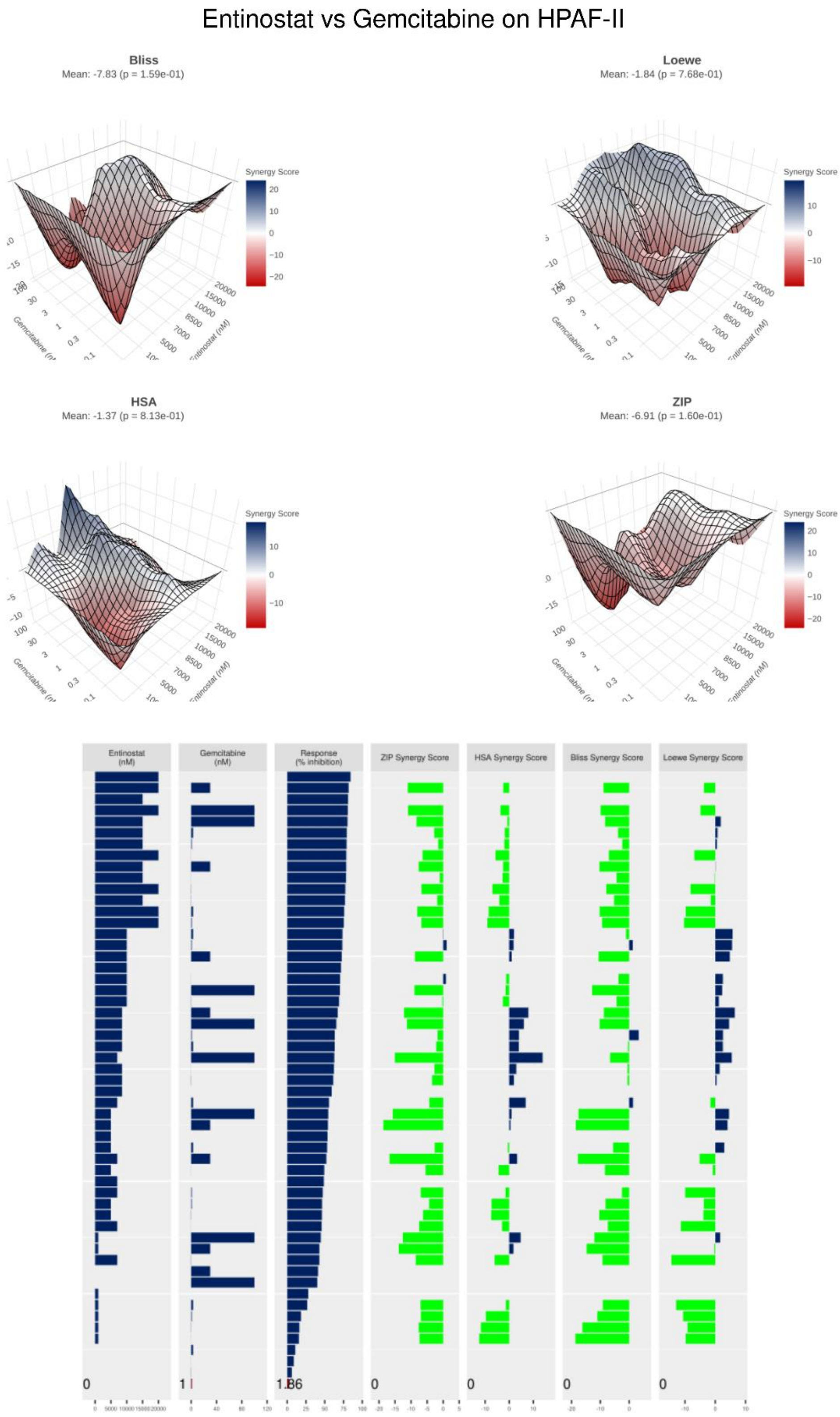

Figure S2: Entinostat vs Gemcitabine synergy on HPAFII cells, Related to Fig. 3A. Bliss, Loewe, HAS and ZIP synergy metrics in 3D as well as bar plot are visualised. Synergy was calculated for three experimental samples.

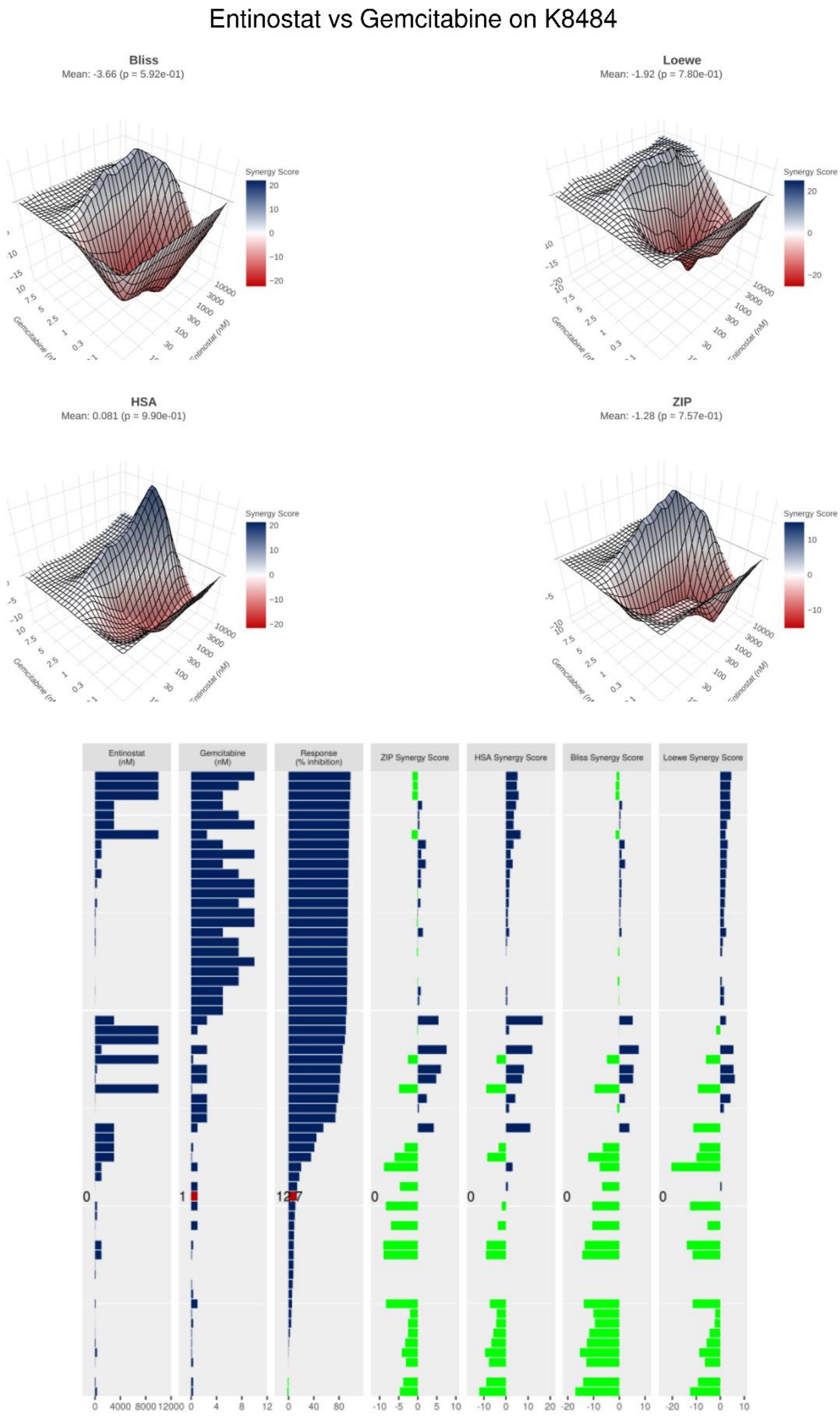

Figure S3: Entinostat vs Gemcitabine synergy on K8484 cells, Related to Fig. 3A. Bliss, Loewe, HAS and ZIP synergy metrics in 3D as well as bar plot are visualised. Synergy was calculated for three experimental samples.

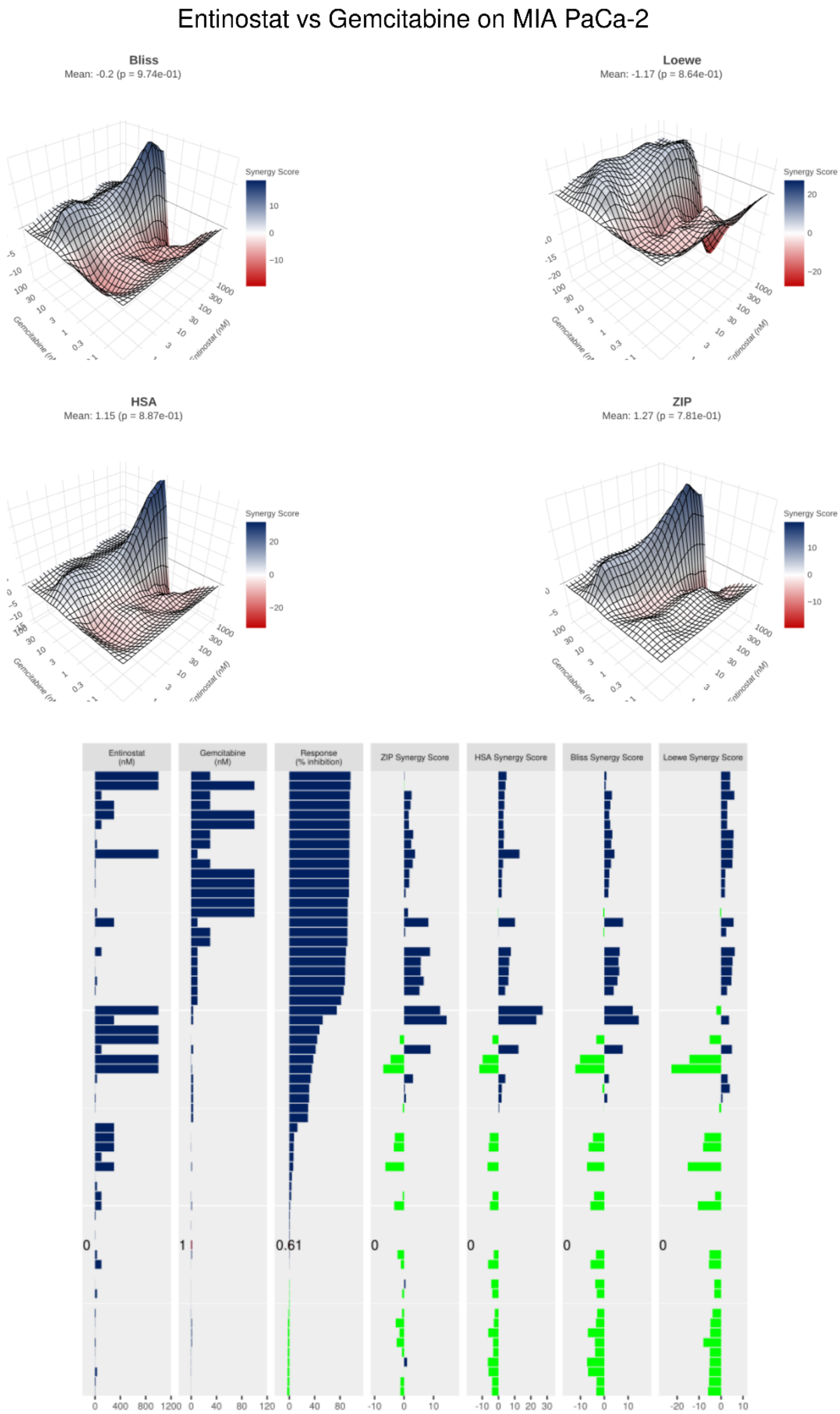

Figure S4: Entinostat vs Gemcitabine synergy on MIA PaCa-2 cells, Related to Fig. 3A. Bliss, Loewe, HAS and ZIP synergy metrics in 3D as well as bar plot are visualised. Synergy was calculated for three experimental samples.

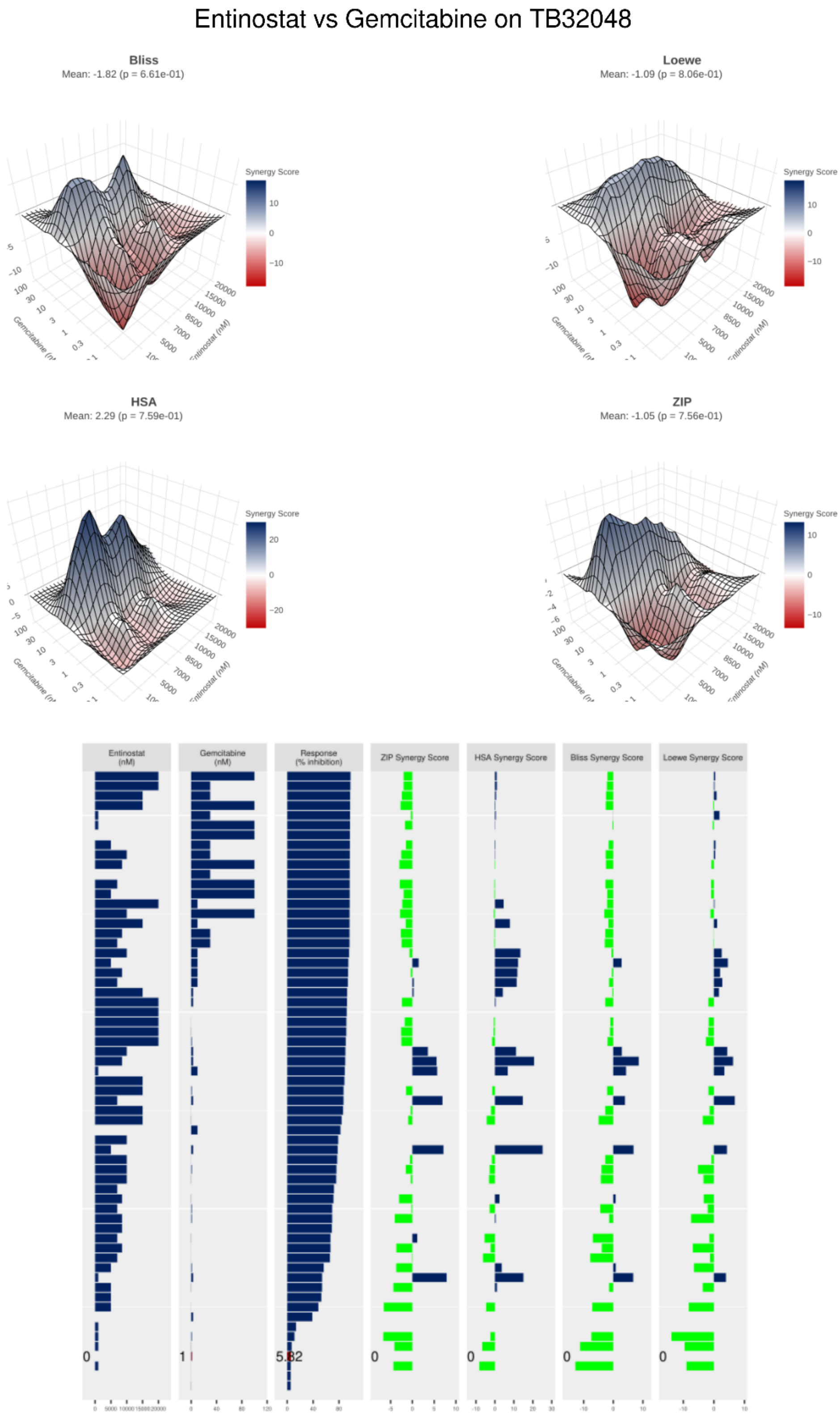

Figure S5: Entinostat vs Gemcitabine synergy on TB32048 cells, Related to Fig. 3A. Bliss, Loewe, HAS and ZIP synergy metrics in 3D as well as bar plot are visualised. Synergy was calculated for three experimental samples.

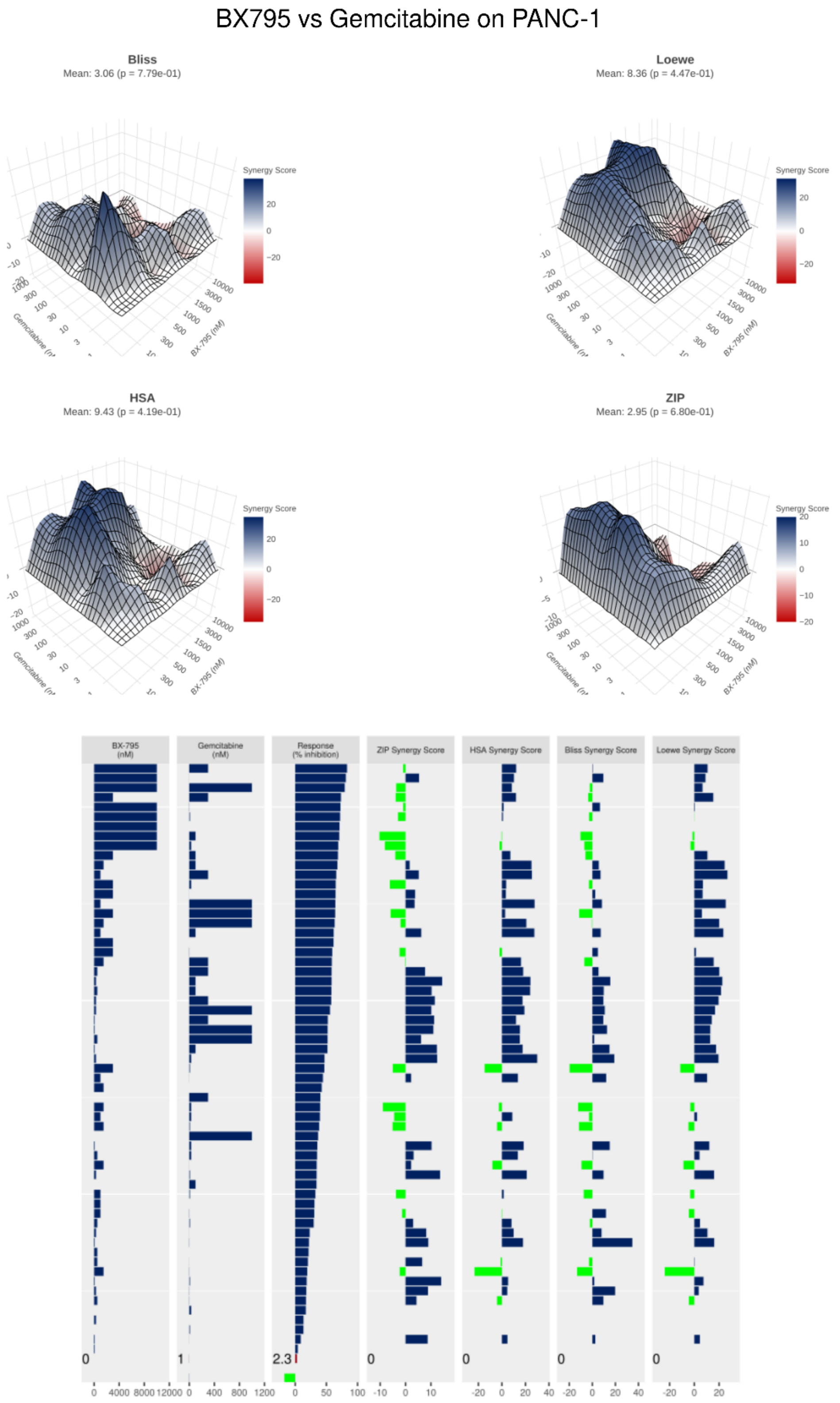

Figure S6: BX795 vs Gemcitabine synergy on PANC1 cells, Related to Fig. 3A. Bliss, Loewe, HAS and ZIP synergy metrics in 3D as well as bar plot are visualised. Synergy was calculated for three experimental samples.

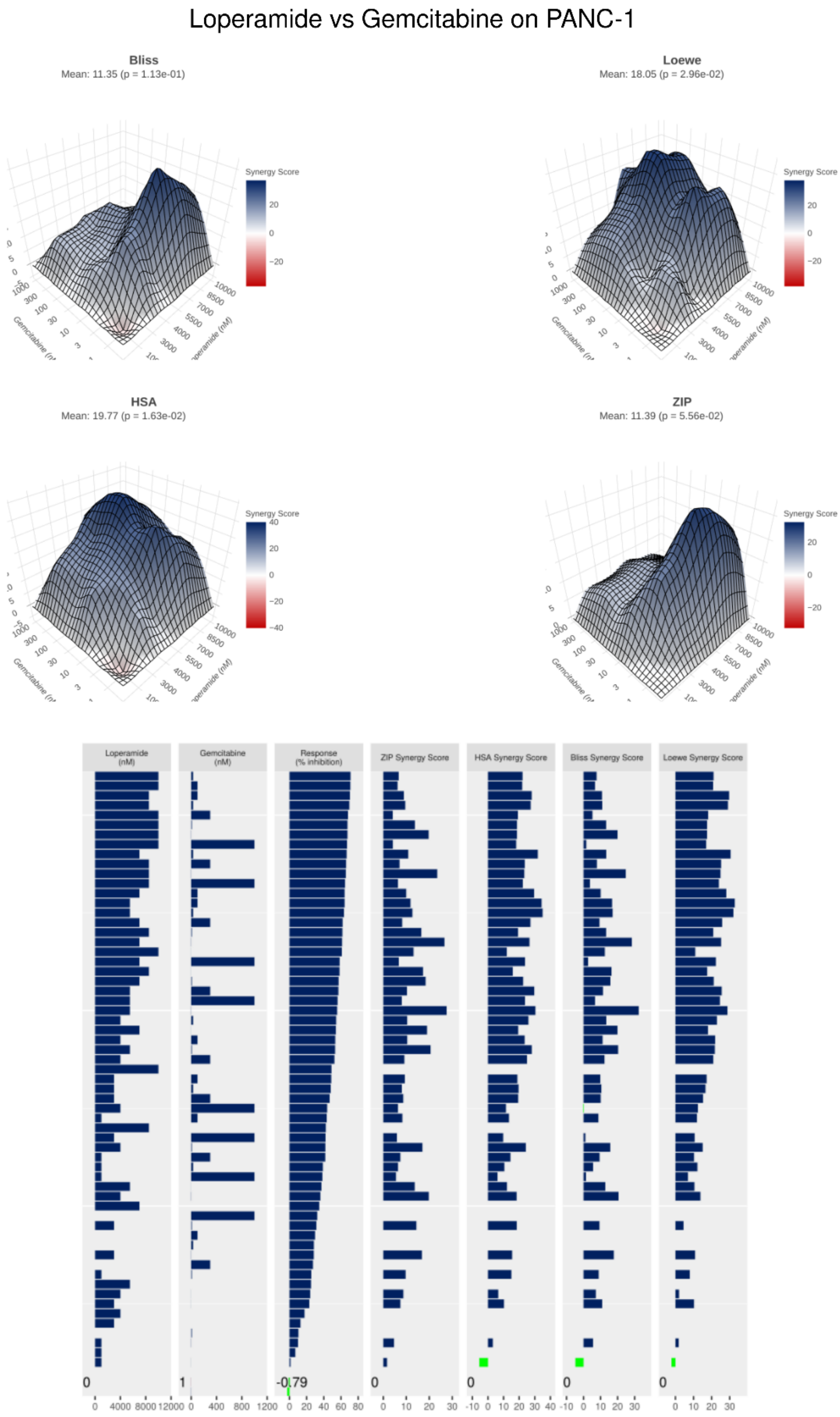

Figure S7: Loperamide vs Gemcitabine synergy on PANC-1 cells, Related to Fig. 3B. Bliss, Loewe, HAS and ZIP synergy metrics in 3D as well as bar plot are visualised. Synergy was calculated for three experimental samples.

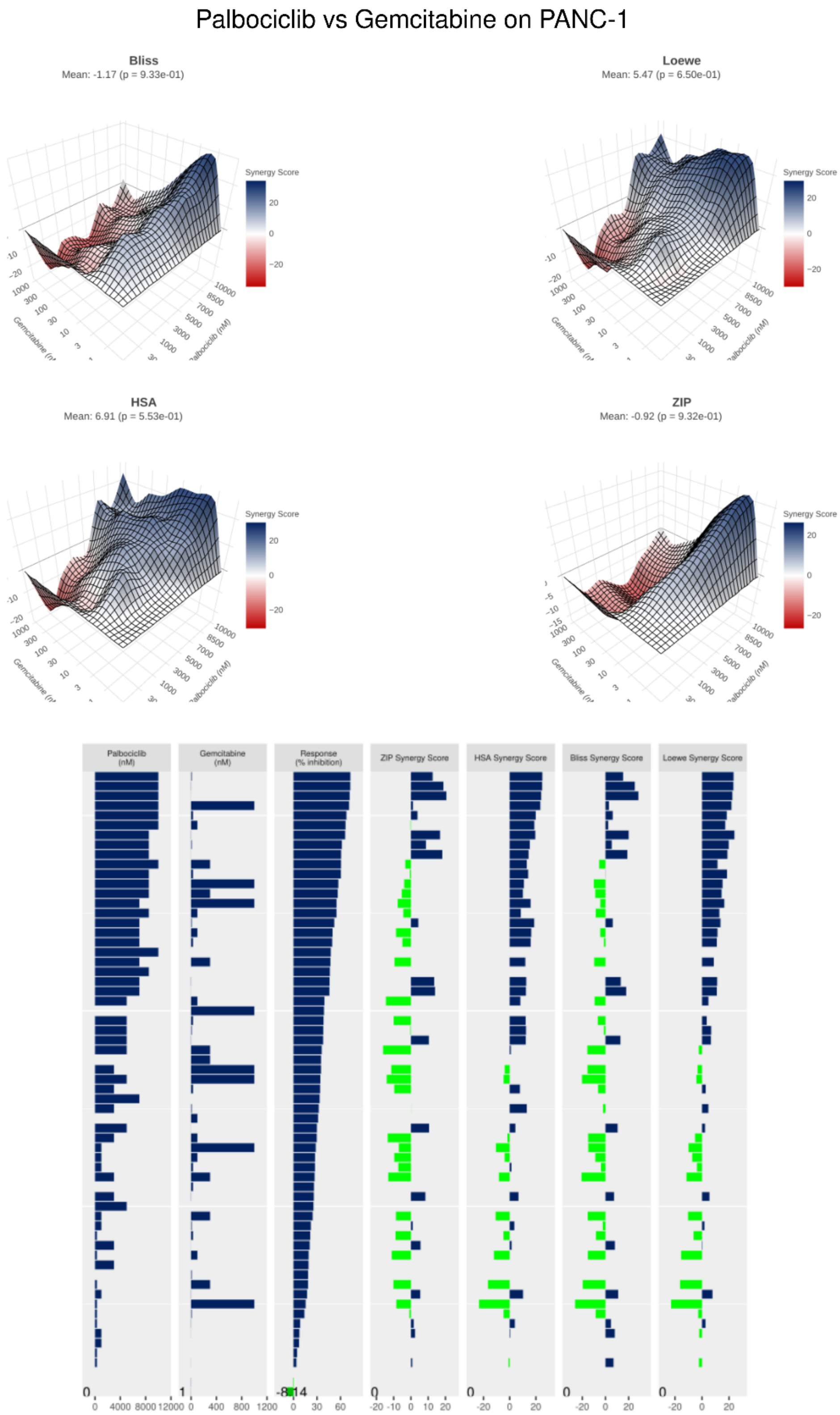

Figure S8: Palbociclib vs Gemcitabine synergy on PANC-1 cells, Related to Fig. 4A. Bliss, Loewe, HAS and ZIP synergy metrics in 3D as well as bar plot are visualised. Synergy was calculated for three experimental samples.

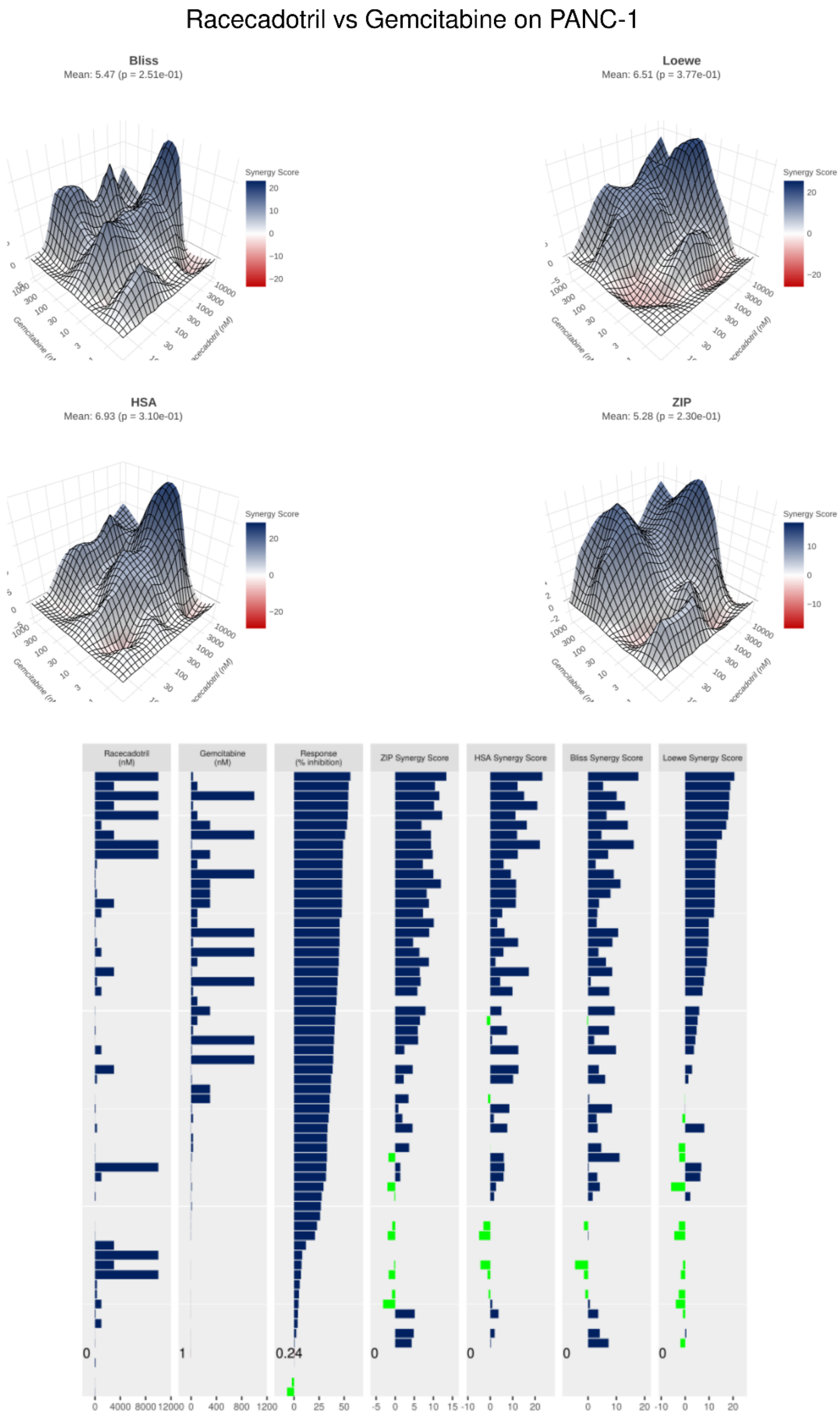

Figure S9: Racecadotril vs Gemcitabine synergy on PANC-1 cells, Related to Fig. 4B. Bliss, Loewe, HAS and ZIP synergy metrics in 3D as well as bar plot are visualised. Synergy was calculated for three experimental samples.

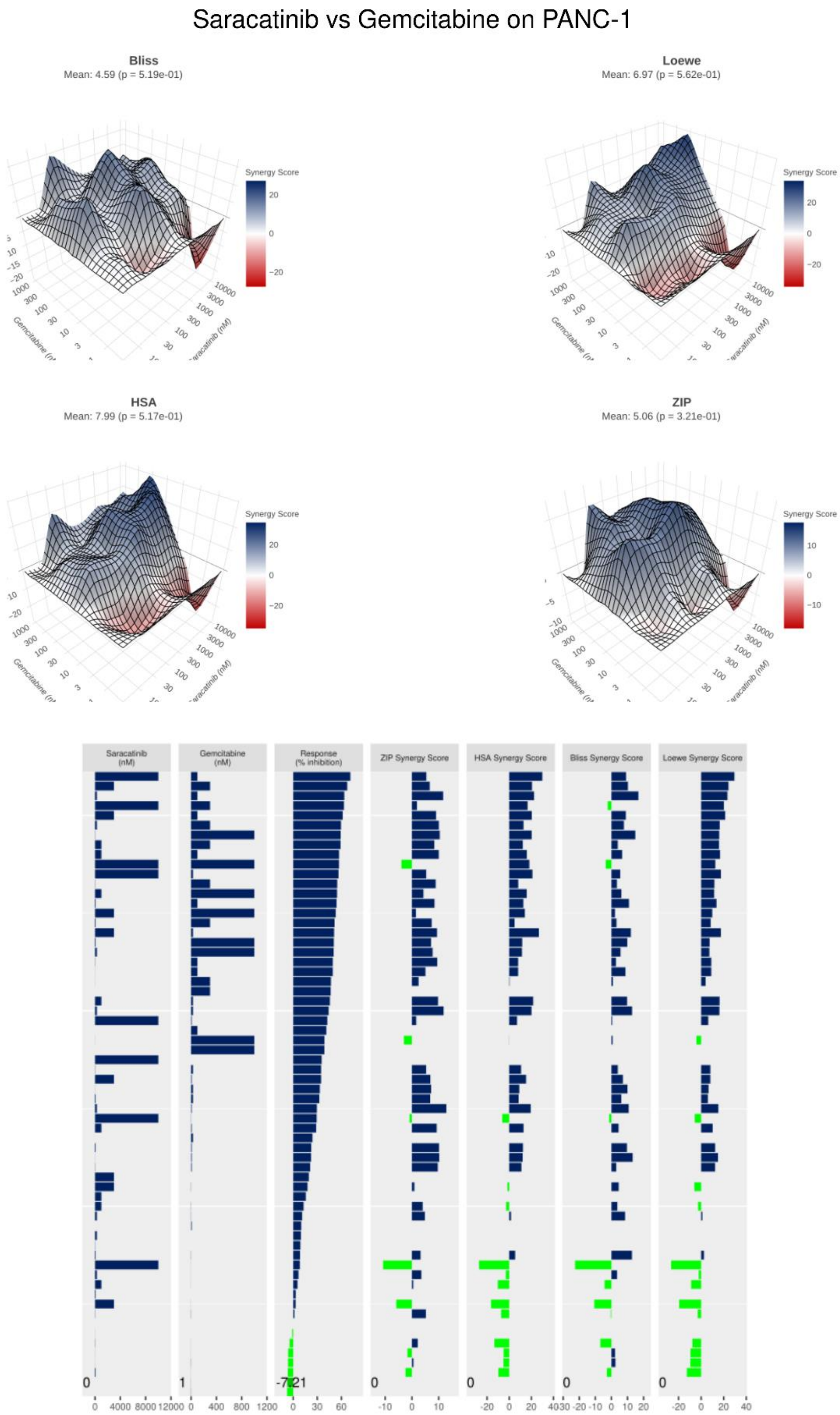

Figure S10: Saracatinib vs Gemcitabine synergy on PANC-1 cells, , Related to Fig. 3D. Bliss, Loewe, HAS and ZIP synergy metrics in 3D as well as bar plot are visualised. Synergy was calculated for three experimental samples.

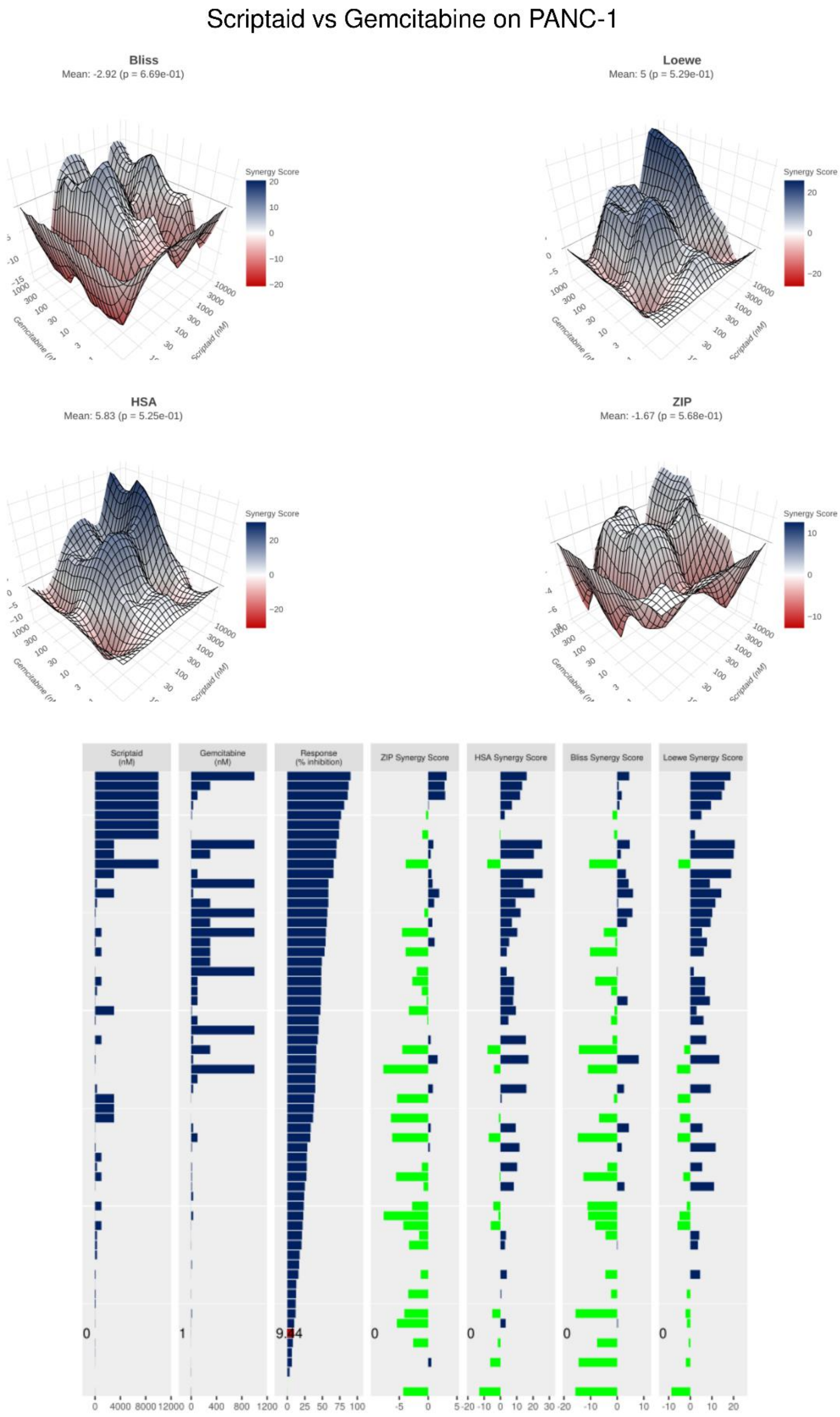

Figure S11: Scriptaid vs Gemcitabine synergy on PANC-1 cells, Related to Fig. 3E. Bliss, Loewe, HAS and ZIP synergy metrics in 3D as well as bar plot are visualised. Synergy was calculated for three experimental samples.

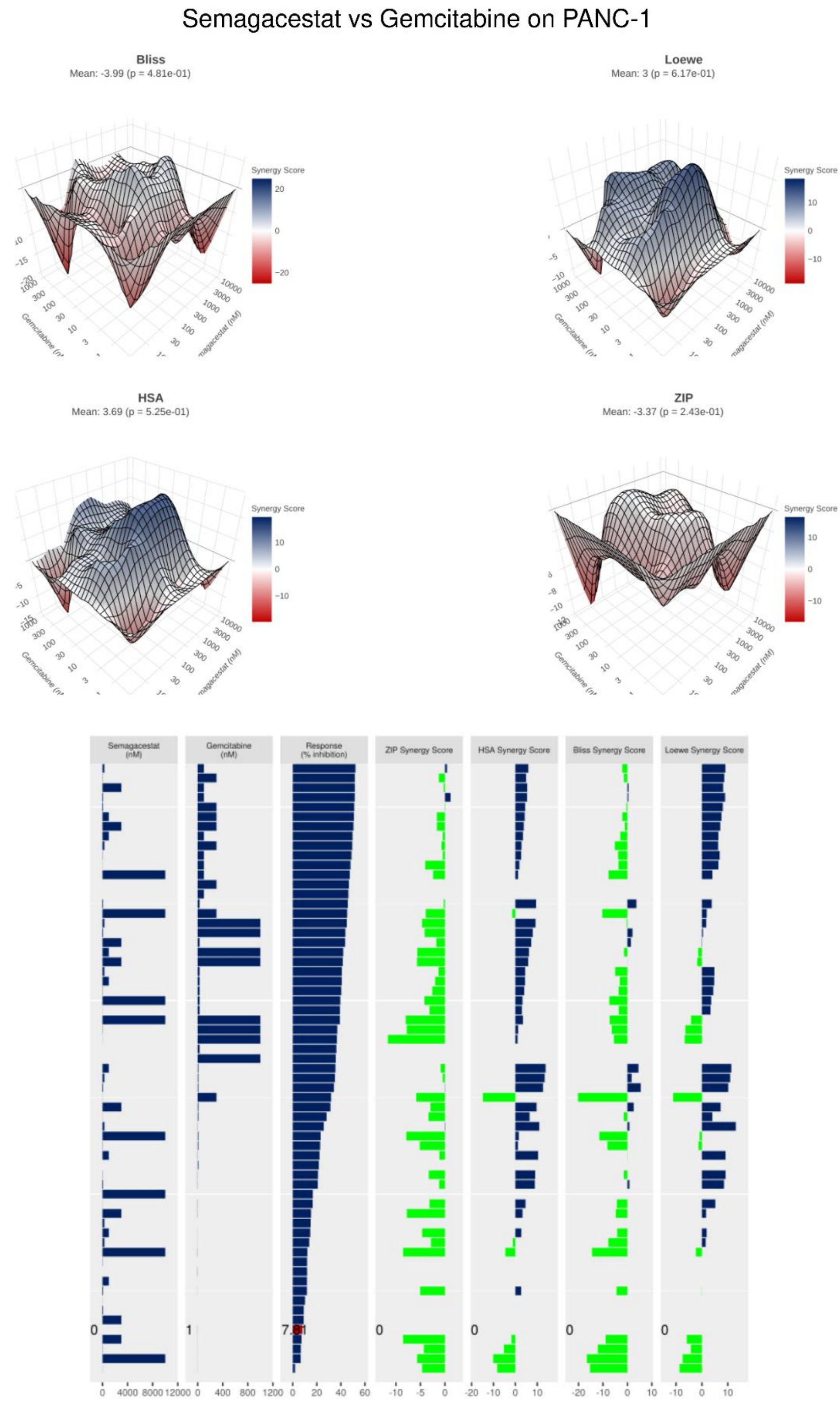

Figure S12: Semagacestat vs Gemcitabine synergy on PANC-1 cells, Related to Fig. 4E. Bliss, Loewe, HAS and ZIP synergy metrics in 3D as well as bar plot are visualised. Synergy was calculated for three experimental samples.

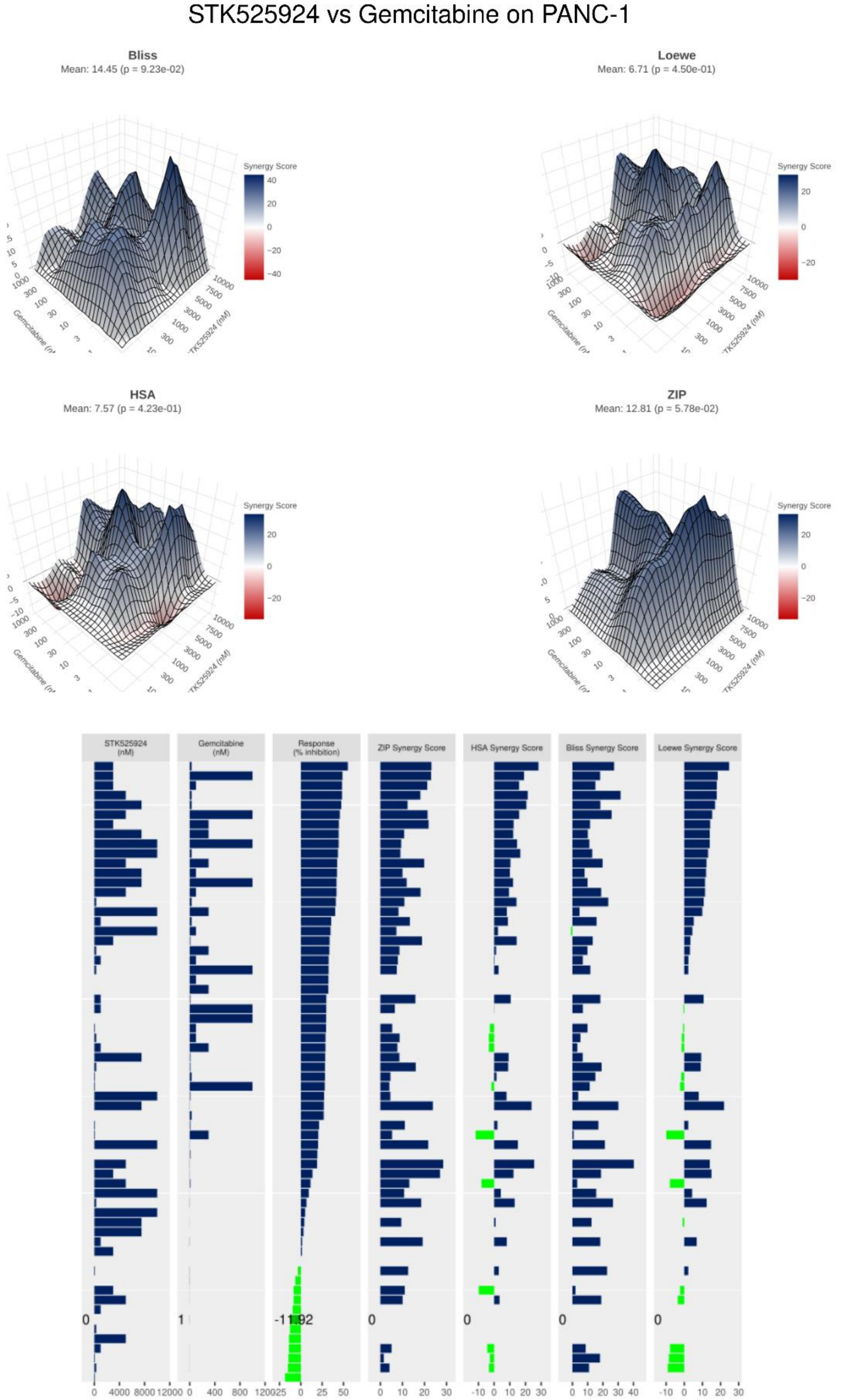

Figure S13: STK525924 vs Gemcitabine synergy on PANC-1 cells, Related to Fig. 4C. Bliss, Loewe, HAS and ZIP synergy metrics in 3D as well as bar plot are visualised. Synergy was calculated for three experimental samples.

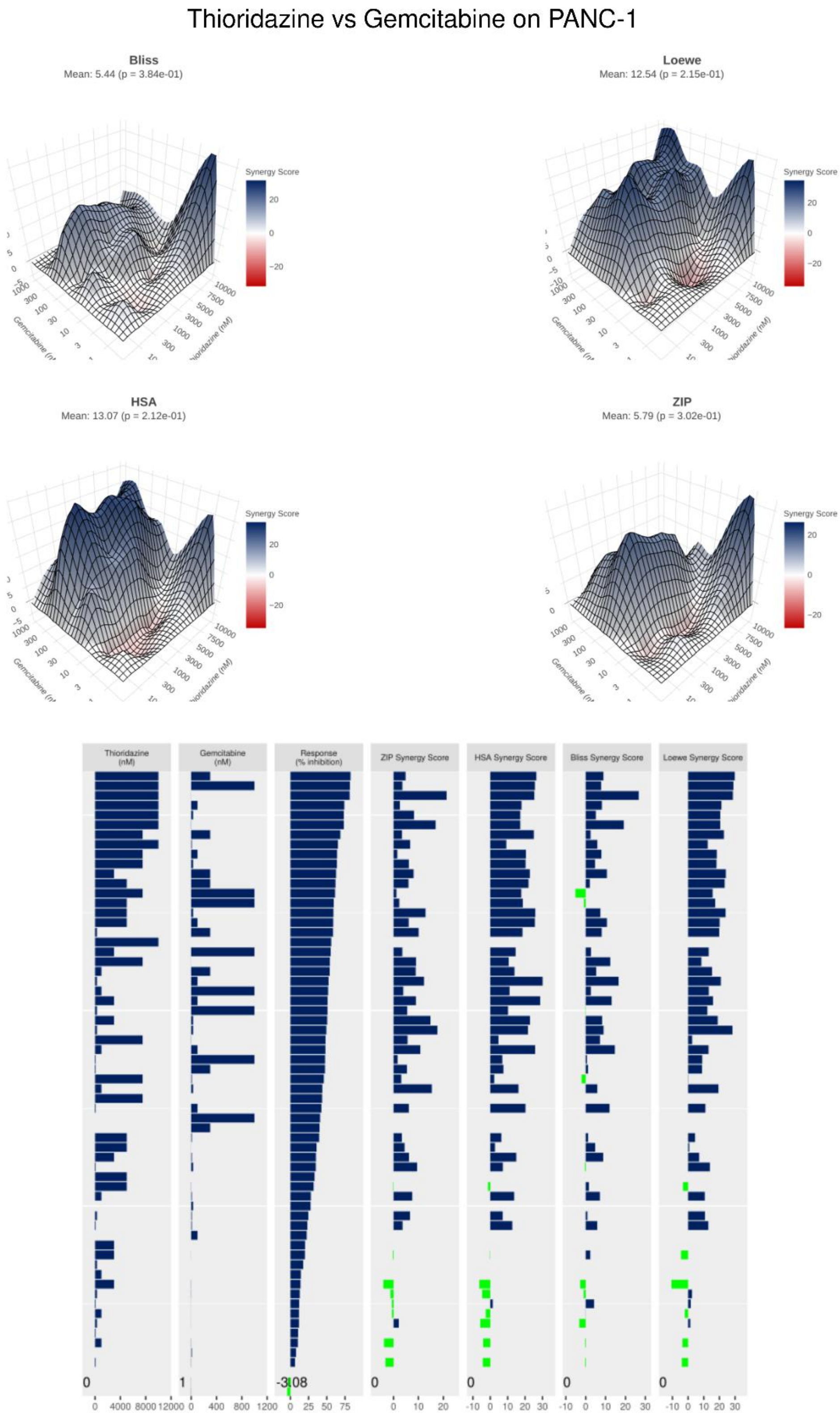

Figure S14: Thioridazine vs Gemcitabine synergy on PANC-1 cells, Related to Fig. 3C. Bliss, Loewe, HAS and ZIP synergy metrics in 3D as well as bar plot are visualised. Synergy was calculated for three experimental samples.

A

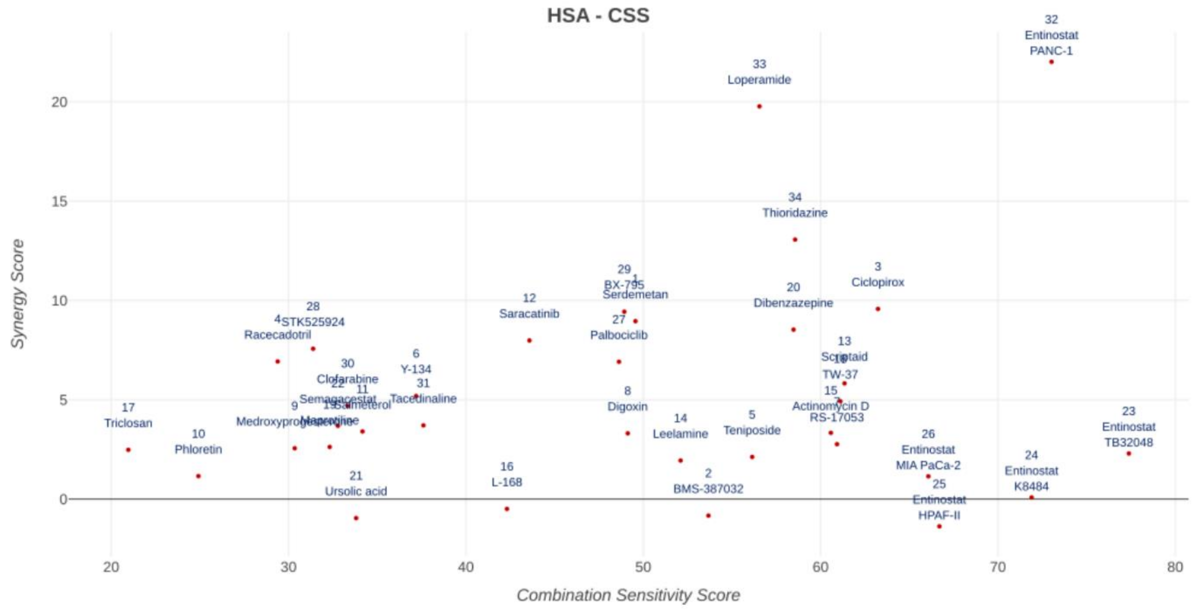

B

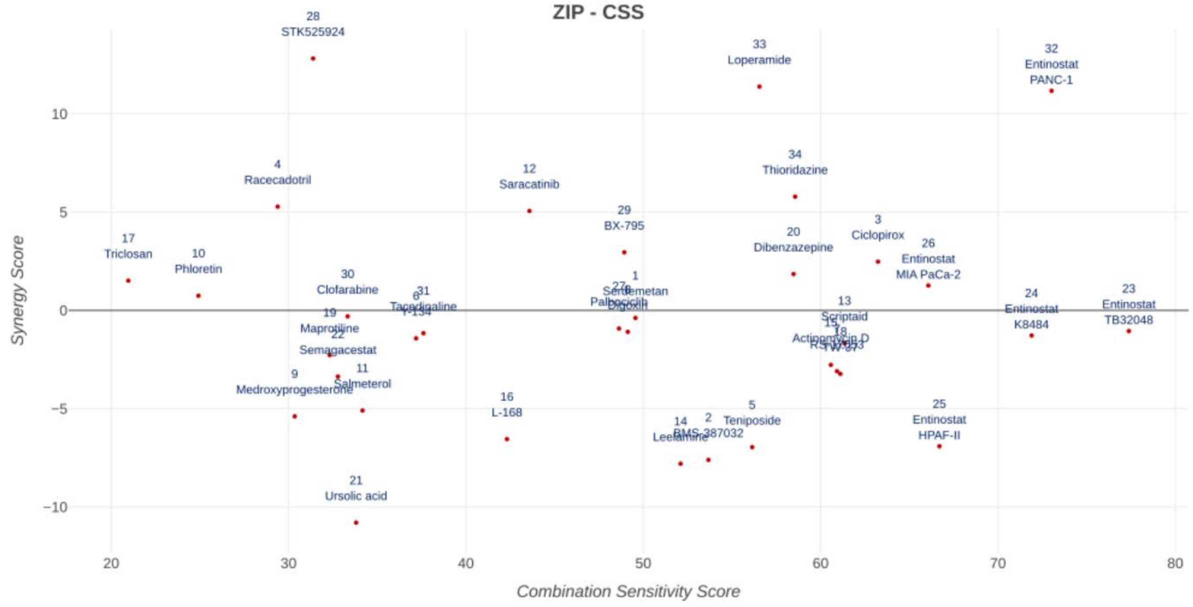

Figure S15: Synergy metrics vs Cell Sensitivity for all compound pairs on PANC-1, Related to Fig. 5. A) HAS and **B)** ZIP synergy metrics are depicted for all experimentally validated compound combinations. Y axis is the synergy metric and x axis is the combination Score sensitivity. Synergy was calculated for three experimental samples.

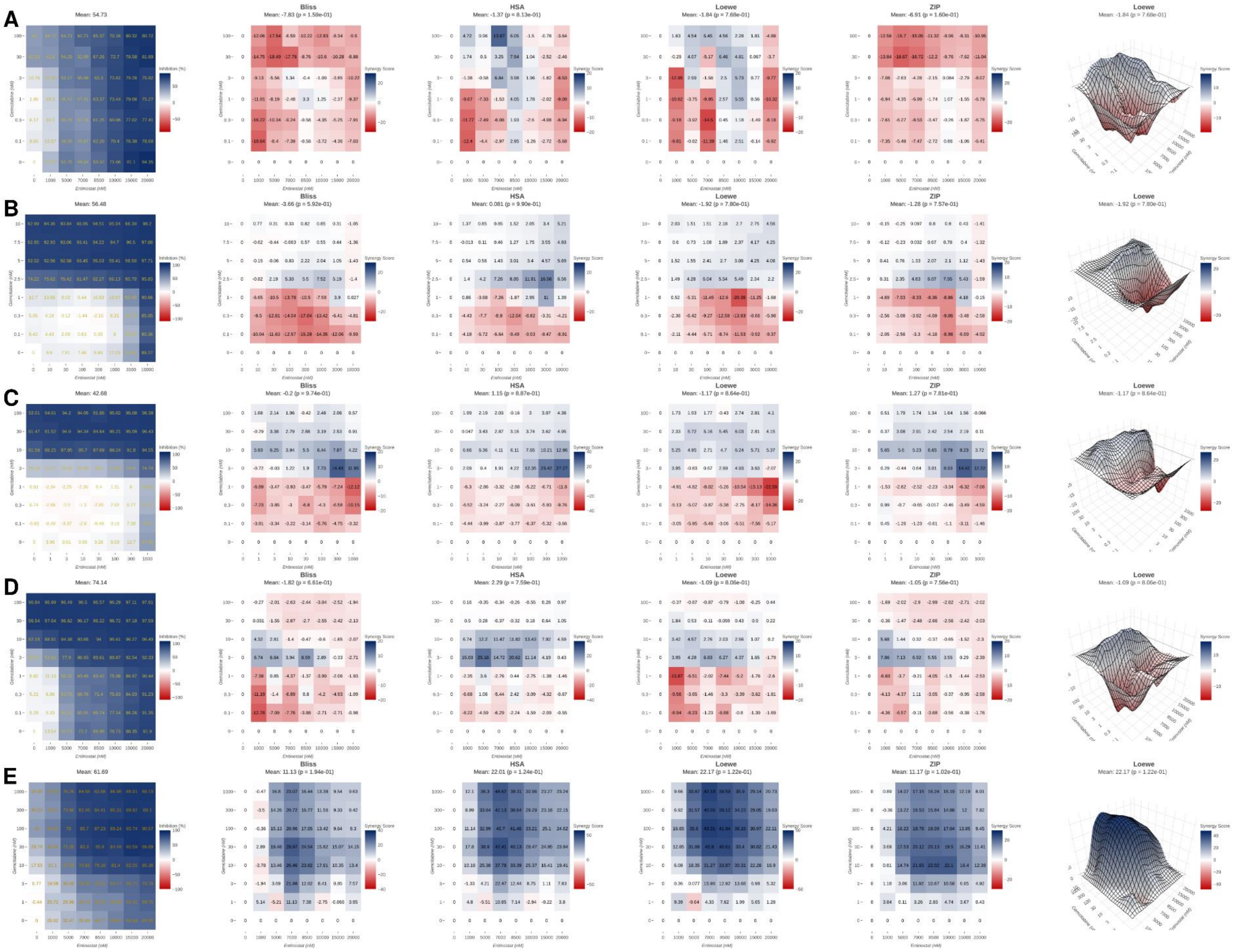

Figure S16: Entinostat-Gemcitabine on 5 pancreatic cancer cell lines, Related to Fig. 3A. From left to right columns: Cell Viability, Bliss, HAS, Loewe, ZIP Synergy metrics in 2D and Loewe Synergy metric in 3D on **A)** HPAF-II, **B)** K8484, **C)** MIA PaCa-2 **D)** TB32048 and **E)** PANC-1 cell lines are depicted. Mean of cell viability for three samples are presented. Synergy was calculated for three experimental samples.

Table S1: ACP Pathways, related to Fig 2A. Anti-correlated Pathways (ACP) between gemcitabine and PANC-1 cell line. Pathway names and BioSystems IDs are provided accompanied with z-score for gemcitabine instance 1 and PANC-1 cell line.

Pathway Name	ID	gemcitabine	PANC1
PLK1 signaling events	138007	-10.01	8.54
Mitotic prometaphase	105815	-9.94	12.19
Resolution of sister chromatid cohesion	730315	-9.65	11.21
M Phase	105812	-9.48	6.8
Kinesins	187197	-8.98	7.5
Mitotic G2-G2/M phases	160942	-7.75	5.16
G2/M transition	105801	-7.75	5.23
Regulation of PLK1 activity at G2/M transition	771569	-7.65	4.68
Separation of sister chromatids	730318	-7.47	7.16
Mitotic anaphase	105818	-7.47	6.89
Mitotic metaphase and Anaphase	730317	-7.47	6.88
Cell cycle Mitotic	105765	-7.44	7.81
Cell cycle	530733	-7.31	8.76
Phosphorylation of the APC/C	105830	-6.65	3.92
APC/C:Cdc20 mediated degradation of Cyclin B	105832	-6.65	3.43
Activation of NIMA kinases NEK9 NEK6 NEK7	771571	-6.6	6.78
Phosphorylation of emi1	105827	-6.59	6.14
Golgi cisternae pericentriolar stack reorganization	105814	-6.59	3.35
Cyclin A/B1 associated events during G2/M transition	105802	-6.42	3.07
Gastric cancer network 2	760637	-5.95	11.75
Polo-like kinase mediated events	105806	-5.9	8.46
Centrosome maturation	105807	-5.9	5.05
Recruitment of mitotic centrosome proteins and complexes	105808	-5.9	5.05
AhR pathway	755436	5.47	-3.14
Condensation of prometaphase chromosomes	730316	-5.39	8.87
FOXM1 transcription factor network	137935	-5.38	9.09
Aurora B signaling	138080	-5.2	9.83
Regulation of beta-cell development	106328	5.13	-3.27
Gastric cancer network 1	760635	-5.03	7.84
Loss of proteins required for interphase microtubule organizationÂ from the centrosome	105810	-4.68	3.55
Loss of Nlp from mitotic centrosomes	105811	-4.68	3.55
Pancreatic secretion	169306	4.46	-5.43
Selenium pathway	198825	4.42	-7.7
Aldosterone-regulated sodium reabsorption	130626	4.4	-2.92
Maturity onset diabetes of the young	83096	4.4	-3.57
Hedgehog signaling pathway	198835	-3.86	3.59
Osteopontin-mediated events	137960	3.56	-2.94
GPCR ligand binding	161020	3.33	-2.94
Integrins in angiogenesis	137999	3.2	-10.82
Osteopontin signaling	198761	3.16	-5.33
Recruitment of NuMA to mitotic centrosomes	105809	-3.15	4.81
Osteoclast Signaling	198769	3.14	-3.97
DEx/H-box helicases activate type I IFN and inflammatory cytokines production	833822	3.13	-4.53
Synthesis of prostaglandins (PG) and thromboxanes (TX)	730333	3.13	-4.63
Factors involved in megakaryocyte development and platelet production	187196	-2.96	3.73
FOXA2 and FOXA3 transcription factor networks	137911	-2.93	-7.08
Cytokine-cytokine receptor interaction	83051	2.69	-3.09
Class A/1 (Rhodopsin-like receptors)	106357	2.66	-3.09
Folate metabolism	198833	2.59	-6.8

Table S2: CP Pathways, related to Fig 2B-E. Corelated Pathways between gemcitabine instances and PANC-1 cell line are listed here with Biosystems IDs. Each pathway that contributed to each scoring system is identified.

**Pathway Name**	**ID**	**Score Type**
Notch signalling pathway	169345	Score1/ Res-score
Superpathway of steroid hormone biosynthesis	907943	Score1/ Res-score
Calcineurin-regulated NFAT-dependent transcription in lymphocytes	137993	Score1
Chromosome maintenance	161048	Score1
Metabolism	477135	Score1
FOXA1 transcription factor network	137979	Score2
MAPK targets/ Nuclear events mediated by MAP kinases	160138	Score2/ Res-score
TGF Beta signaling pathway	198810	Score2
Signaling by activin	730349	Score2
Chromosome maintenance	161048	Selected
Folate metabolism	198833	Selected
Hedgehog signaling pathway	198835	Selected
Fructose and mannose metabolism	82930	Selected
Superpathway of steroid hormone biosynthesis	907943	Selected
MAPK targets/ Nuclear events mediated by MAP kinases	160138	Selected
Aurora B signaling	138080	Selected

Table S3: Compounds predicted to exhibit synergy in combination with gemcitabine with existing literature support (see main text for references), Related to Table 1. Scores are in the range -1 to 1 with scores close to -1 for single agents meaning being active as single agents and scores close to -1 for combination meaning the compound is predicted to be synergistic. (Single agent scores are generally lower compared to combination scores as single agent scores are calculated based on anticorrelation of larger number of pathways and scores for combination are calculated based on anticorrelations on just a few specific pathways that were systematically hypothesized to be related to gemcitabine resistance.)

**Compound**	**Combination Score Type**	**Combination score**	**Score as a single agent**
Berbamine	Score1	-0.889	-0.139
Masitinib		-0.763	-0.259
Gossypol	Score2	-0.663	-0.18
Menadione		-0.65	-0.176
Triptolide	Res-score	-0.982<br>-0.968 (for another instance)	-0.132
Panobinostat		-0.988	-0.106
Belinostat		-0.982	-0.117
Fluvastatin		-0.957	-0.312
Trichostatin-A		-0.968	-0.185

Table S4: Compound Signatures, Related to STAR Methods. Top 50 up/down regulated genes of entinostat, gemcitabine and trichostatin-A retrieved from LINCS database are listed here. Genes in bold are members of chromosome maintenance pathway that are down regulated in entinostat signature but up regulated in the gemcitabine signature (instance 1).

Compound sig	Genes
Entinostat_down CPC013_MCF7_24H:BRD-K77908580-001-04-7:10	**BRCA1**,NFKBIB,AURKA,LSM5,MYCBP,GATA3,GLOD4,CDK7,ADI1,PRR7,ARPP19,SLC35B1,TIMELESS,PHF15,**HSD17B10**,**RFC5**,KIF2C,UBE2C,MCM3,**LIG1**,CDC25A,TCERG1,TP53,PLK1,**POLE2**,CDC45,CDK4,TRAPPC3,SPAG7,RNF167,MRPL12,ATF5,PCBD1,RRS1,ICAM3,SUV39H1,DPH2,CDC20,IFRD2,NUP85,BIRC5,PUF60,PARP2,XBP1,CCNA2,NUP88,TIMM9,**PCNA**,BDH1,SPDEF
Entinostat_up CPC013_MCF7_24H:BRD-K77908580-001-04-7:10	FOXO4,TMEM2,CEBPA,CD97,GLRX,STXBP1,COL1A1,NFIL3,HSD17B11,MYO10,KDM5B,NPC1,INSIG1,GNB5,LGMN,RGS2,ALDOC,LIPA,CYTH1,SPP1,STX1A,P4HA2,CTSL1,WFS1,BLVRA,ID2,ATP1B1,PSMB8,IGF2BP2,KIAA1033,EPB41L2,RAB31,PLS1,SERPINE1,PGM1,CALU,KIF5C,POLD4,TSPAN3,BNIP3L,NCOA3,S100A4,ECH1,CSRP1,HMGCS1,CDKN1A,CDH3,ST3GAL5,IGFBP3,CCNA1
Gemcitabine instance1_down CPC006_A375_6H:BRD-K15108141-001-01-7:0.08	SH3BP5,IARS2,KIAA1033,HADH,HSD17B11,CCNH,WIPF2,MRPL19,TP53BP1,SUZ12,RNMT,SMC4,CHMP4A,CENPE,ELAC2,CYCS,TUBB6,MBNL1,LBR,BUB1B,CDC25B,TLE1,NUP93,NCOA3,KIAA0528,CCNF,TLK2,ACOT9,CCNB2,HSPA1A,TES,PCM1,TP53BP2,CDC20,PAN2,RFX5,ITGAE,PSRC1,TOP2A,AURKB,CCDC86,KIF2C,KIF14,KIF20A,CCNB1,AURKA,PLK1,TXNDC9,POLG2,UBE2C
Gemcitabine instance1_up CPC006_A375_6H:BRD-K15108141-001-01-7:0.08	EGR1,JUN,CDKN1A,SRC,FAS,SESN1,GADD45A,ZFP36,NFATC3,NFKB2,FOS,BLCAP,ARID5B,HES1,**RFC2**,DDB2,**PCNA**,SFN,EZH2,KIAA0355,**RPA2**,EVL,**LIG1**,BCL7B,DUSP14,TBXA2R,PDGFA,MCM3,IER3,EED,RHOA,GNB5,PLSCR3,CYB561,CDK5R1,PTGS2,NOTCH1,FHL2,ST3GAL5,CERK,PMAIP1,KAT6B,GATA2,CCL2,FAM57A,ZDHHC6,SPP1,ATP1B1,DRAP1,MMP1
Gemcitabine instance2_down RAD001_MCF7_24H:BRD-K15108141-003-01-3:0.3704	TIMM9,TSKU,SPDEF,SMNDC1,GPATCH8,COG4,GLOD4,RRP1B,TMEM109,SMARCD2,VGLL4,FBXO7,GABPB1,LRPAP1,TPM1,DDX42,SYPL1,STAT1,CAMSAP2,PWP1,CSK,CDC25B,GRWD1,ABL1,RRP12,NOSIP,TXNRD1,PAK4,BIRC5,MIF,AURKA,PLK1,MTA1,PAF1,KIF14,CCDC86,KIF2C,PSRC1,ATF1,TOP2A,TARBP1,OXSR1,IFRD2,CDC20,CCNB2,KIF20A,UBE2C,TEX10,OXA1L,GTF2E2
Gemcitabine instance2_up RAD001_MCF7_24H:BRD-K15108141-003-01-3:0.3704	RRS1,VAV3,SPAG4,HPRT1,FGFR2,SNAP25,P4HA2,CDKN1A,ERBB3,CCL2,RAB21,PTK2,HOXA5,CRKL,FOSL1,IQGAP1,IL1B,KIT,ORC1,IKBKB,PRKCQ,POLR1C,DDIT4,CD44,PEX11A,INPP1,SOCS2,CBR1,MOK,ZNF318,DHRS7,MKNK1,GFOD1,NPRL2,SLC11A2,PIK3CA,ACAT2,MYCBP2,WDR67,MMP1,COL4A1,UBE2L6,PTGS2,RPA2,HIST2H2BE,CSNK2A2,DDB2,SLC25A46,SMC1A,CCNH
Trichostatin-A_up HOG003_MCF7_24H:BRD-K68202742-001-10-8:0.3704_true	FOXO4,ALDOC,HMOX1,TRAK2,GLRX,PLA2G15,INSIG1,STX1A,PSMB8,NPC1,STXBP1,TMEM2,COL1A1,HDAC6,SQSTM1,TCTA,ACLY,GRN,FYN,GNAI2,PIK3R3,NFIL3,TSPAN3,POLD4,HSD17B11,FGFR4,DDIT4,CCND3,MAN2B1,BNIP3L,RGS2,CYTH1,HMGCS1,PHKG2,EDEM1,ATP6V1D,GNB5,KDM5B,HIST1H2BK,LIPA,TXNRD1,MYO10,CCDC92,PTPN1,PRAF2,SSBP2,EPHB2,WDTC1,SPAG4,MEF2C
Trichostatin-A_down HOG003_MCF7_24H:BRD-K68202742-001-10-8:0.3704_true	KEAP1,SUV39H1,SMNDC1,ITGB5,GABPB1,KAT6B,ERBB3,NET1,TMEM97,ATF5,MTHFD2,CDC25A,IFRD2,PRR7,MRPL19,SYPL1,RRP1B,NPDC1,PHF15,INPP4B,DPH2,ITGAE,IGF1R,SORBS3,SCCPDH,ADI1,CDK4,FAM57A,CDC20,CCND1,MRPL12,PRSS23,NUP88,**POLE2**,BIRC5,SPR,PCBD1,HSPA8,SPAG7,**PCNA**,UBE2C,FHL2,NPEPL1,BDH1,PUF60,CCNA2,XBP1,MYC,SPDEF,TP53
